# Spatial regulation of a common precursor from two distinct genes generates metabolite diversity†
†Electronic supplementary information (ESI) available. See DOI: 10.1039/c5sc01058f



**DOI:** 10.1039/c5sc01058f

**Published:** 2015-07-13

**Authors:** Chun-Jun Guo, Wei-Wen Sun, Kenneth S. Bruno, Berl R. Oakley, Nancy P. Keller, Clay C. C. Wang

**Affiliations:** a Department of Pharmacology and Pharmaceutical Sciences , School of Pharmacy , University of Southern California , Los Angeles , CA 90089 , USA . Email: clayw@usc.edu; b Chemical and Biological Process Development Group , Energy and Environment Directorate , Pacific Northwest National Laboratory , Richland , WA 99352 , USA; c Department of Molecular Biosciences , University of Kansas , Lawrence , KS 66045 , USA; d Department of Medical Microbiology and Immunology , University of Wisconsin-Madison , Madison , WI 53706 , USA; e Department of Chemistry , College of Letters, Arts, and Sciences , University of Southern California , Los Angeles , CA 90089 , USA

## Abstract

In secondary metabolite biosynthesis, core synthetic genes such as polyketide synthase genes usually encode proteins that generate various backbone precursors. These precursors are modified by other tailoring enzymes to yield a large variety of different secondary metabolites. The number of core synthesis genes in a given species correlates, therefore, with the number of types of secondary metabolites the organism can produce. In our study, heterologous expression of all the *A. terreus* NRPS-like genes showed that two NRPS-like proteins, encoded by *atmelA* and *apvA*, release the same natural product, aspulvinone E. In hyphae this compound is converted to aspulvinones whereas in conidia it is converted to melanin. The genes are expressed in different tissues and this spatial control is probably regulated by their own specific promoters. Comparative genomics indicates that *atmelA* and *apvA* might share a same ancestral gene and the gene *apvA* is located in a highly conserved region in *Aspergillus* species that contains genes coding for life-essential proteins. Our data reveal the first case in secondary metabolite biosynthesis in which the tissue specific production of a single compound directs it into two separate pathways, producing distinct compounds with different functions. Our data also reveal that a single *trans*-prenyltransferase, AbpB, prenylates two substrates, aspulvinones and butyrolactones, revealing that genes outside of contiguous secondary metabolism gene clusters can modify more than one compound thereby expanding metabolite diversity. Our study raises the possibility of incorporation of spatial, cell-type specificity in expression of secondary metabolites of biological interest and provides new insight into designing and reconstituting their biosynthetic pathways.

## Abstract 2

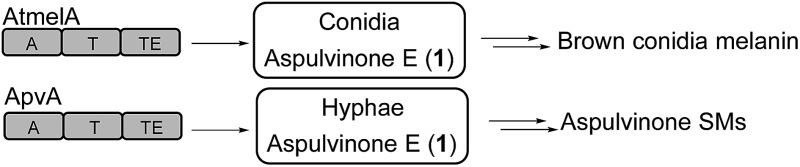
We have demonstrated that spatial regulation of the same product from two distinct genes generates metabolite diversity.

## Introduction

Filamentous fungi, such as species found within the genus *Aspergillus*, are well known producers of secondary metabolites (SMs) with interesting biological activities. The genome sequencing of *Aspergillus* species revealed that the number of putative SM genes greatly exceeds the number of identified SMs, suggesting many novel types of SMs still remain to be discovered.^1^ In general, these SM genes are clustered in the genome.^2^ A typical cluster contains one key gene required for the synthesis of the precursor skeleton. These genes usually encode large multidomain enzymes belonging to the polyketide synthase (PKS) or non-ribosomal peptide synthetase (NRPS) class.^3^ Other adjacent genes encode enzymes that are involved in tailoring modifications, transport of the product, or co-regulation of the cluster genes.^4^ In a given fungal species, the SM arsenal is usually governed by the number of key synthetic genes in the genome, and this diversity is multiplied by various tailoring enzymes that give rise to a considerable number of different natural products.^5^ For instance, the fungus *A. nidulans* has 27 PKS genes, 12 NRPS genes, 14 NRPS-like genes and one PKS/NRPS hybrid gene.^6^ In a previous study, Ahuja *et al.* systematically characterized the polyketide products of eight non reducing PKSs (NR-PKSs) in *A. nidulans*.^5^ In combination with the six previously characterized NR-PKS genes, the study demonstrated that the 14 NR-PKS genes in *A. nidulans* could be divided into seven groups based on phylogenetic analysis. More importantly, each of these NR-PKSs produces a unique product which can be modified by other tailoring enzymes and incorporate into various SM biosynthetic pathways, indicating the potential of *A. nidulans* for biosynthesizing a large variety of different PKS-derived SMs.^5^

Recently, application of an efficient gene targeting system enabled us to link two NRPS-like genes *apvA* and *btyA* to their corresponding SMs, aspulvinones and butyrolactones, respectively, in *A. terreus* strain NIH 2624.^7^ In this study, we demonstrated that one NRPS-like gene, *atmelA*, is involved in the synthesis of a brown conidial melanin in this fungus.^7^ Compared to typical NRPSs, NRPS-like genes encode single module (A-T-TE) proteins missing the condensation (C) domain.^3,8^ The adenylation (A) domain is responsible for aryl acid substrate recognition and activation. The activated substrate is loaded onto the thiolation (T) domain. The thioesterase (TE) domain is suggested to be involved in the condensation and releasing of the final product.^8^ Therefore, the diversity of NRPS-like products is expanded by a combination of different A domains (substrate selection and activation) and TE domains (various cyclization and release mechanisms). The *A. terreus* genome contains 14 NRPS-like genes with predicted A-T-TE or similar domain architecture and the SM products for the majority of these are unknown.

**Fig. 1:**
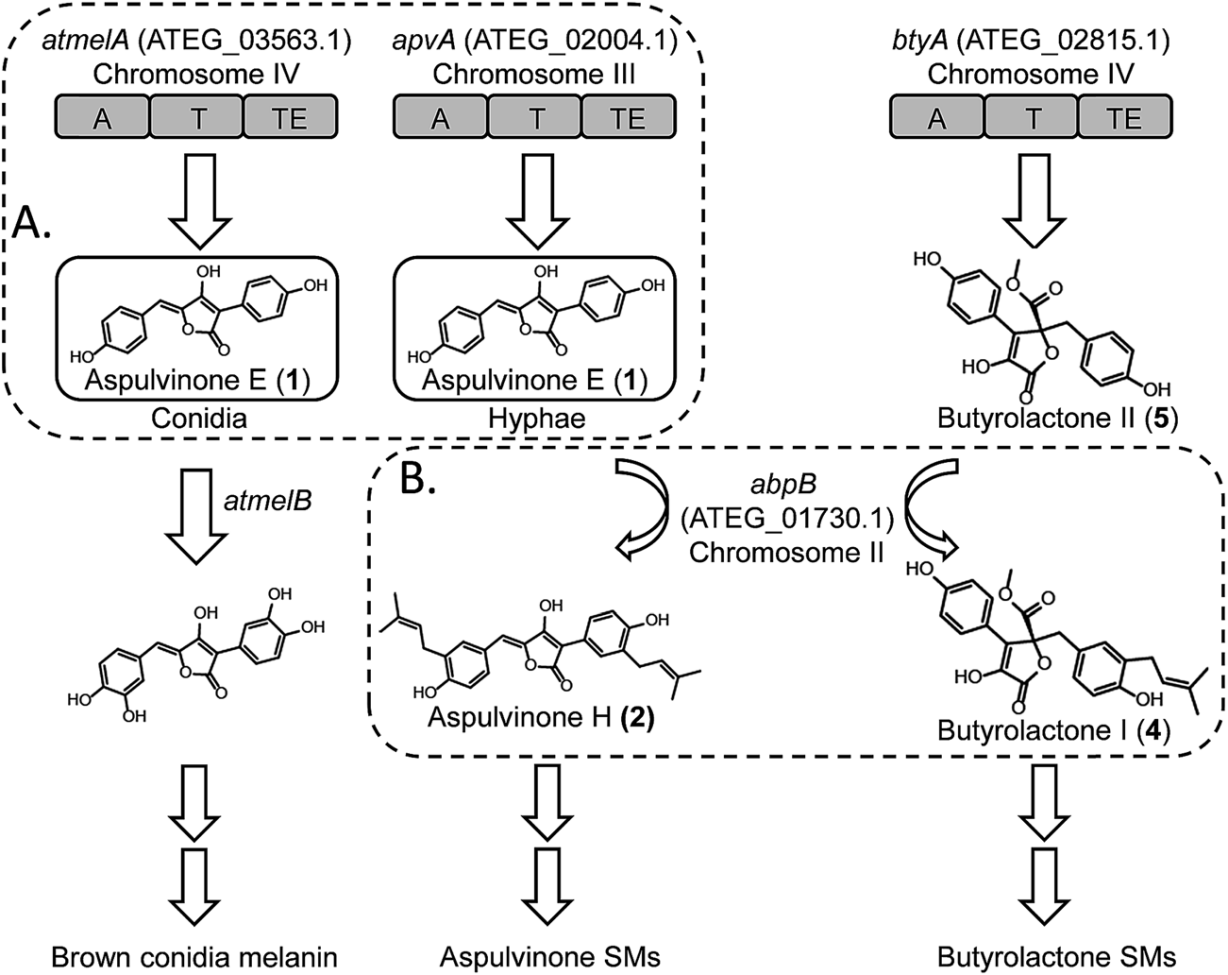
(A) AtmelA and ApvA may synthesize the identical natural product **1** accumulated in different fungal tissues. (B) A *trans*-prenyltransferase, AbpB, prenylates two substrates, aspulvinones and butyrolactones.

Here we report our efforts to systematically characterize the products of these 14 NRPS-like genes. We heterologously expressed each gene in *A. nidulans* under the control of the inducible *alcA* promoter. Surprisingly, our study reveals that two NRPS-like genes, *apvA* and *atmelA*, are responsible for the formation of the same intermediate, aspulvinone E (**1**, Fig. 1A). The aspulvinone E produced by AtmelA is further modified by a tyrosinase, AtmelB, and is incorporated into the brown melanin biosynthesis pathway in *A. terreus* (Fig. 1A). In parallel, the aspulvinone E synthesized by ApvA is further prenylated by a *trans*-prenyltransferase (AbpB) to produce aspulvinones (Fig. 1B). AbpB also prenylates butyrolactones, and this reveals that modifying genes outside of specific clusters can be responsible for modifying compounds from more than one SM gene cluster, thus expanding the diversity of SMs produced by an organism (Fig. 1B).

Our results suggest that the promoter of *apvA* drives expression in hyphae resulting in the production of aspulvinone E (**1**), which is modified to produce aspulvinone variants. The *atmelA* promoter drives expression in conidia, also resulting in the production of aspulvinone E (**1**) but in this cell type it is converted to melanin. Further genetic analysis of *apvA* and *atmelA* indicates that these two genes may share a common ancestral gene. The gene *apvA*, which may result from duplication of the ancestral gene, is inserted in a genomic region consisting of genes that codes for life-essential proteins. Our study suggests an unprecedented pathway for conidial pigment biosynthesis in *A. terreus* that incorporates an NRPS-like product aspulvinone E (**1**) as its substrate (Fig. 1). Our data also demonstrated how the SM diversity can be expanded by (1) allocating the same natural product in different fungal compartments to produce molecules with different functions; (2) encoding tailoring enzyme that is capable of chemically modifying more than one type of SMs.

## Results

### Heterologous expression of the NRPS-like genes *apvA* and *atmelA* in *A. nidulans* both result in aspulvinone E production

**Fig. 2:**
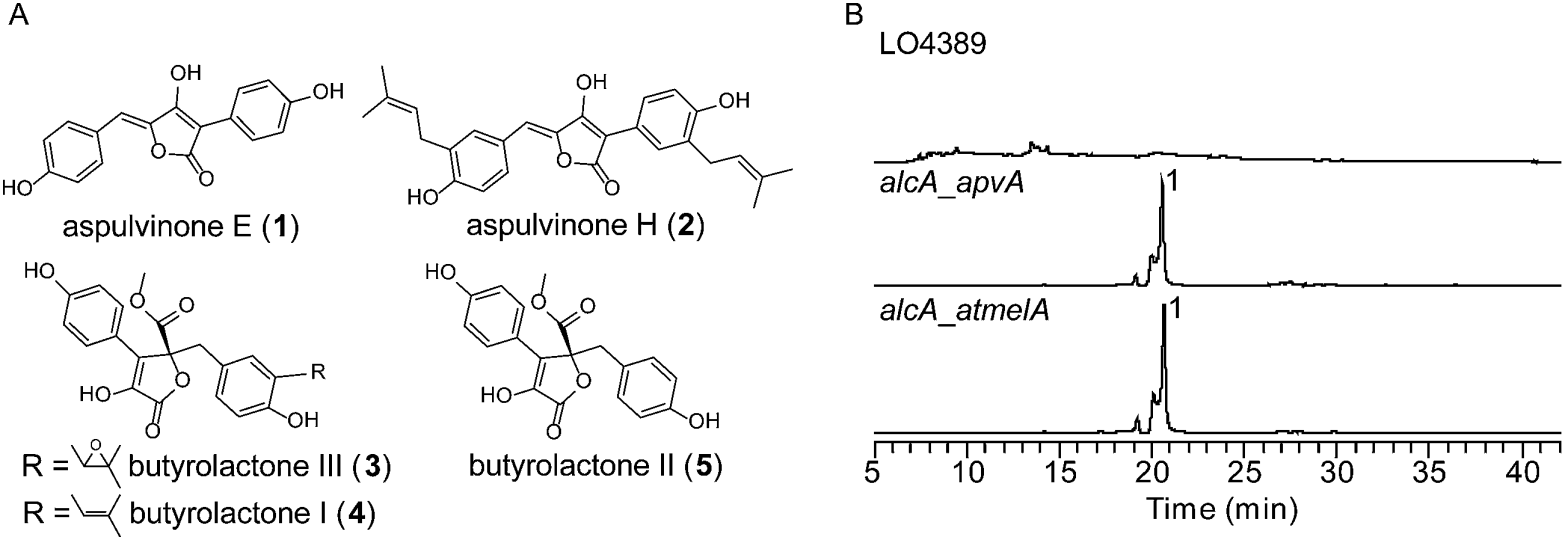
(A) Compounds related to this study. (B) HPLC profiles of extracts of HE strains as detected by total scan UV.

**Fig. 3:**
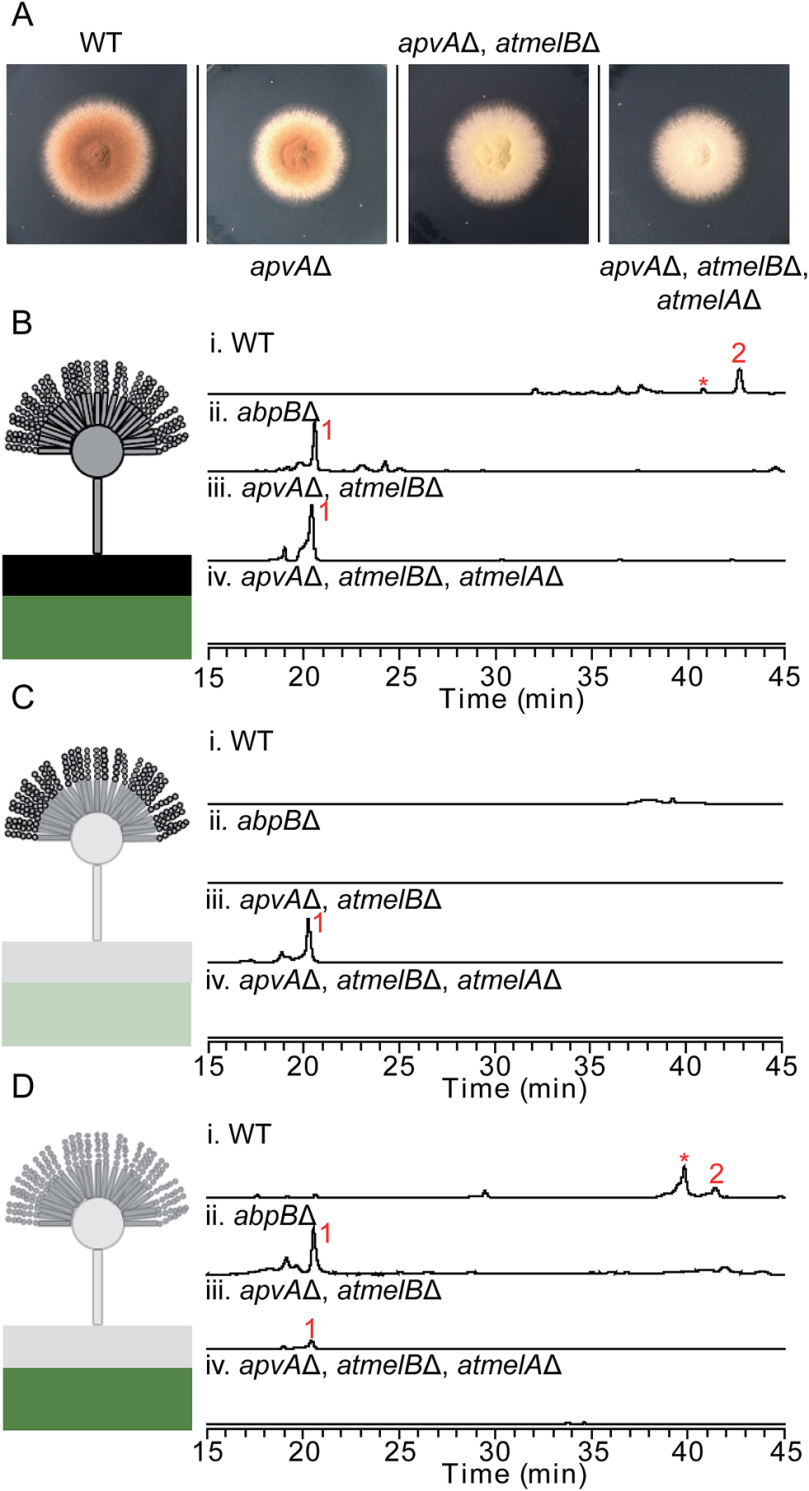
(A) Phenotype of *A. terreus* wild type and other *A. terreus* mutant strains growing on LCMM for 5 days. Morphological change of the fungal conidia can be observed if the deleted genes are involved in the biosynthesis of the brown conidial pigment. Total extracts (B), conidial extracts (C) and hyphal extracts (D) of *A. terreus* wild type and mutant strains as detected by UV at 370 nm. Aspulvinone related natural products are labeled in red. In B–D, the black box represents the top layer agar as described in tissue-specific extraction. The green box represents the bottom layer agar in tissue-specific extraction. The numbering of the peaks corresponds to the natural products shown in Fig. 2A. The aspulvinone E (**1**) and its related natural products can be detected in LC/MS traces Bi, Bii, Biii, Ciii, Di, Dii, and Diii. Trace amount of **1** identified in D iii is due to the diffusion of this compound synthesized in the conidia of the mutant strain. *This metabolite is related with aspulvinones according to its UV absorption and MS spectrum.

Using a recently reported efficient heterologous expression (HE) system in *A. nidulans*,^9^ the individual NRPS-like genes identified in the *A. terreus* genome were expressed at either the *wA* or *yA* locus of *A. nidulans* under regulation of the inducible *alcA* promoter (Fig. S1A†). In a previous study, targeted deletion of *apvA* depleted production of aspulvinones in *A. terreus* indicating that this NRPS-like gene is responsible for the biosynthesis of the aspulvinone core.^7^ As expected, heterologous expression of *apvA* results in accumulation of aspulvinone E (**1**) which is speculated to be the first intermediate in the aspulvinone pathway (Fig. 1 and 2A and B).^7^ Unexpectedly, our HE experiments revealed that AtmelA produces the same compound (Fig. 2A and B). The gene *apvA* is responsible for aspulvinone biosynthesis while *atmelA* is involved in the synthesis of the brown conidial pigment.^7^ The brown conidial melanin is still produced in the *apvA* deletant strain as shown in Fig. 3A. In contrast, deletion of *atmelA* generates an albino mutant that is still capable of synthesizing aspulvinones.^7^ Together, these data reveal that despite having the same activity (*i.e.* synthesis of aspulvinone E), ApvA and AtmelA function in different roles in the fungus.

### Aspulvinone E occurs as a precursor in both aspulvinone and melanin pathways

**Fig. 4:**
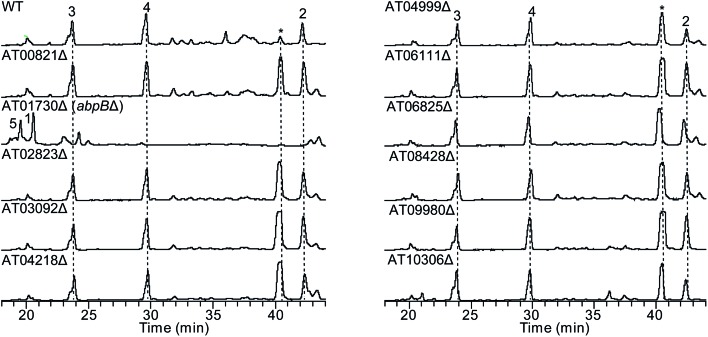
HPLC profiles of extracts of *A. terreus* prenyltransferase (PT) genes deletants as detected by UV at 330 nm and 370 nm. The numbering of the peaks corresponds to the natural products shown in Fig. 2A. Compounds **3** and **4** are prenylated derivatives of compound **5**. The “*” compound is an aspulvinone derivative according to its UV absorption spectrum. ATXXXXX is abbreviated for “ATEG_XXXXX.1”. Deletion of the gene ATEG_00702.1, a homolog of *tdiB* involved in the asterriquinone biosynthesis, was not achieved due to the unsuccessful PCR amplification of its flanking region.

To test our hypothesis that the aspulvinone E is an intermediate in two different pathways, the aspulvinone pathway and melanin pathway, we wished to delete the first tailoring gene in each pathway to accumulate the precursor produced by the gene responsible for the backbone metabolite. Since we have established the genetic linkage between *apvA* and aspulvinones,^7^ we set out to identify the first tailoring enzyme, which we presumed was responsible for prenylating **1** to give **2** (Fig. 2A). However, we could not locate a prenyl transferase (PT) gene proximal to *apvA*.^7^ We then targeted each of the putative PT genes in the *A. terreus* genome for deletion (Fig. 4). The individual genes were knocked out using fragments created by fusion PCR.^7^ The SM profiles of the correct mutants were examined by LC-MS, and of the 11 different mutants, only the ATEG_01730.1Δ strain accumulated aspulvinone E (**1**) (Fig. 4). Unexpectedly, removal of ATEG_01730.1 leads to the accumulation of butyrolactone II (**5**) as well (Fig. 4). A previous study showed that the NRPS-like gene *btyA* is responsible for the biosynthesis of butyrolactone core.^7^ The expression of three genes, *apvA*, *btyA*, and *abpB* were also analyzed using real-time quantitative reverse transcription PCR (qRT-PCR). (Fig. S2C†) Our data showed that these three genes are co-expressed under aspulvinone and butyrolactone producing conditions. These pieces of evidence suggest that this single PT is responsible for the prenylation of two different metabolites, aspulvinone E (**1**) and butyrolactone II (**5**). Thus, we name the gene *a* (aspulvinone) *b* (butyrolactone) *p* (PT) *B*.

Definitively establishing the role of AtmelA in the synthesis of aspulvinone E (**1**) required additional gene deletion experiments. We first deleted the gene *apvA* using the direct repeat (DR) strategy^10^ followed by the recycling of the *AfpyrG* marker (Fig. S1B†). This ensures that aspulvinone E (**1**) identified in the later mutants is not from ApvA. However, limited information is available about the biosynthesis of the brown conidial melanin in *A. terreus*. Our study suggests that the melanin pathway originates from the NRPS-like product aspulvinone E (**1**), which is unlike the two main precursors [di-hydroxyphenylalanin (DOPA) and dihydroxynaphthalene (DHN)] of currently known melanins.^11^ The biosynthesis of DOPA melanin starts with a tyrosine that is oxidized to either DOPA or dopaquinone (DAQ) by a tyrosinase.^11^ Considering that aspulvinone E (**1**) shares the same phenol moiety as tyrosine, we speculate that a similar tyrosinase might catalyze the hydroxylation of **1** at the *ortho* position to give a dihydroxylated intermediate that becomes incorporated into the brown melanin (Fig. 1). Following the cluster paradigm, we examined the genes surrounding *atmelA* and identified one gene ATEG_03564.1 (*atmelB*) that encodes a putative tyrosinase. Removal of this gene in the *apvA*Δ background changed the strain's phenotype: the brown melanin was no longer synthesized and the conidia became bright yellow (Fig. 3A). As expected, the yellow material is aspulvinone E (**1**), which accumulated in the metabolite profiles of the *apvA* and *atmelB* double deletion strain (Fig. 3B). We next deleted the gene *atmelA* in the double *apvA*Δ, *atmelB*Δ strain. The LC-MS profile of this triple deletion mutant shows the abrogation of the aspulvinone E (**1**) which had reappeared in the double mutant (Fig. 3B), in accord with our HE results that AtmelA was also capable of producing Aspulvinone E (**1**).

### Aspulvinone E (**1**) produced by ApvA and AtmelA accumulates in different fungal tissues

Our studies indicate that ApvA and AtmelA synthesize the same product aspulvinone E (**1**). We next asked how the fungus is able to allocate the same chemical synthesized by different proteins into their own specific pathway without cross-interference. Previous literature reports have shown that the production of SMs and/or their precursors can be specific to both cellular organelle and fungal tissue.^12,13^ We speculated that the aspulvinone E (**1**) from the two genes might be generated in different fungal tissues. Since removal of *atmelA* or *atmelB* changes the phenotype of *A. terreus* conidia (Fig. 3A), it is likely that aspulvinone E (**1**) from AtmelA might be produced in conidia. Likewise aspulvinones, derived from **1** that is produced by ApvA, might be produced inside the hyphae and secreted into the medium.

To test this hypothesis, we performed tissue-specific extraction^12,13^ to reinvestigate the SM profiles of the four strains ((1) wild type; (2) *abpB*Δ; (3) *apvA*Δ, *atmelB*Δ; (4) *apvA*Δ, *atmelB*Δ, *atmelA*Δ). Fungal cultures were divided into three fractions: (1) conidial (mostly conidia and minor conidiophore), (2) top layer agar (mixture of conidiophore, vegetative hyphae, minor invasive hyphae), (3) lower layer agar (mostly invasive hyphae) (Fig. 3C and D). Compared to the SM profiles of total extracts (Fig. 3B), extraction of the conidia showed the accumulation of **1** only in the *apvA*Δ, *atmelB*Δ strain (Fig. 3C), not in strains carrying *abpB*Δ or *atmelA*Δ. This result indicates that aspulvinone E (**1**) from AtmelA is produced in conidia. Extraction of the hyphal (mostly invasive) fraction showed the production of **1** in the *abpB*Δ strain and compound **2** in wild type, indicating that aspulvinone E (**1**) from ApvA and its derivative **2** are specifically produced in hyphae.

### Exchanging *atmelA* with *apvA*, under control of the *atmelA* promoter, restores melanin production in *A. terreus*

**Fig. 5:**
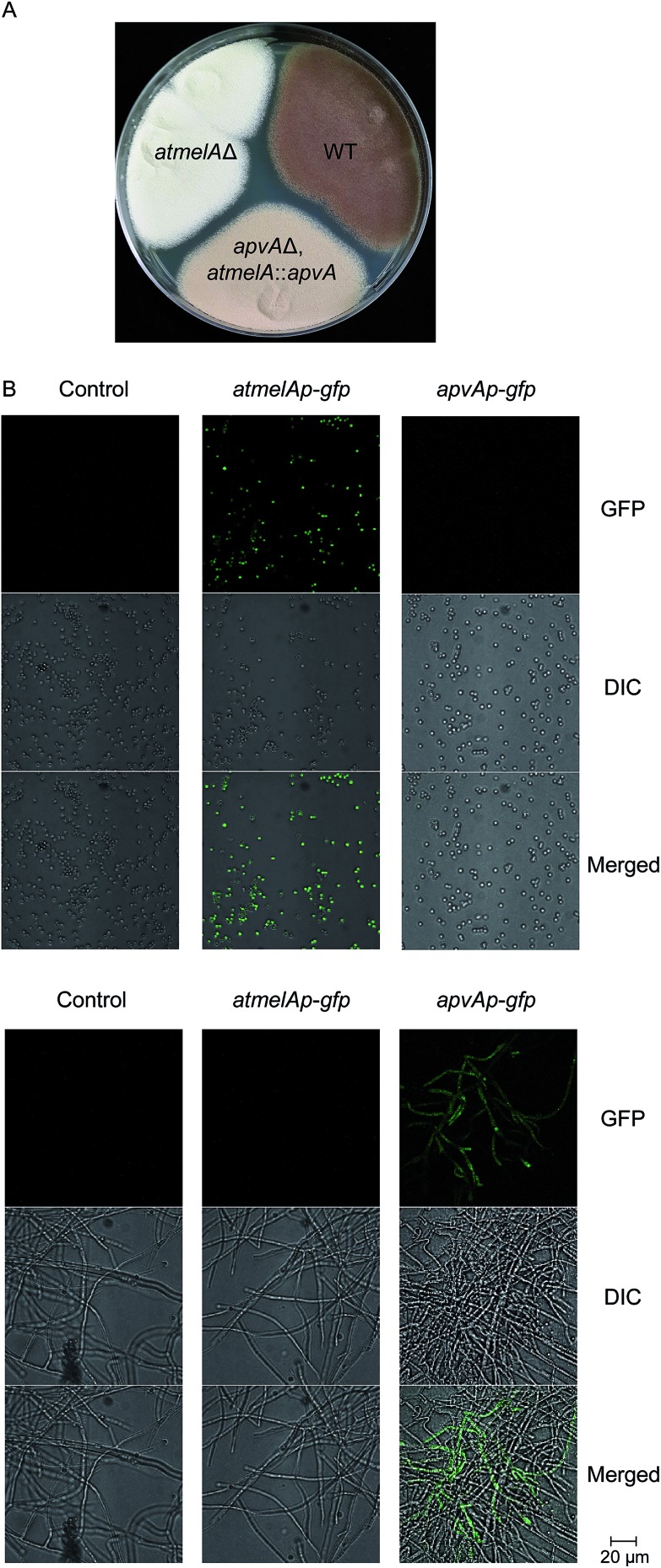
(A) Phenotype of the *A. terreus* mutant strain *apvA*Δ, *atmelA*::*apvA* (the *A. terreus* wild type is used as positive control that produces melanin, the *atmelA*Δ strain is used as negative control). (B) Replacing *atmelA* and *apvA* with *gfp*. Top column: conidia. Bottom column: hyphae. Top row: GFP. Middle row: differential interference contrast (DIC). Bottom row: merged GFP.

Next, we asked about the molecular mechanism for regulating the tissue-specific production of **1**. We first examined the expression profiling of *atmelA* and *apvA* in different tissues using Real-Time qRT-PCR (Fig. S2, see ESI† for experimental details). As expected, the gene *atmelA* is specifically expressed in conidia compartment (Fig. S2B†) while the gene *apvA* is locally expressed in hyphae (Fig. S2C†). We then probed this question by determining whether ApvA could replace AtmelA in brown melanin biosynthesis. We assumed that tailoring enzymes like AtmelB could still recognize aspulvinone E (**1**) generated from either ApvA or AtmelA. Another study suggests that the products of two tubulin genes, *benA* and *tubC*, are functionally interchangeable. The method they used was to disrupt *benA* and then put *tubC* under control of the *benA* promoter.^14,15^ Herein we implemented a similar strategy by replacing the coding region of *atmelA* with that of *apvA*, placing *apvA* under control of the *atmelA* promoter (*atmelAp*) in the *apvA*Δ background (Fig. S1Ci†). As shown in Fig. 5A, the mutant stain (*apvA*Δ, *atmelA*::*apvA*) produces brownish conidia indicating that the brown conidial pigment is produced in the mutant strain (*apvA*Δ, *atmelA*::*apvA*). As anticipated, the production of aspulvinones (**1** or **2**) were not detected (Fig. S3†). The pigment is not produced as much as in the wild type (Fig. 5A) probably because *atmelAp*, after all, is not the native promoter of ApvA. Quantitative analysis of their expression using Real-Time qRT-PCR reveals that the expression level of *apvA*, which is under the control of atmelA promoter, is lower than that of *atmelA*. (Fig. S2B†) This could be one of the reasons for reduced production of the conidial melanin in the mutant strain (*apvA*Δ, *atmelA*::*apvA*). But more importantly, our experiment shows that compound **1** from *apvA* can be incorporated into the melanin pathway when *apvA* is regulated by *atmelAp*. As mentioned earlier, the production of **1** from *atmelA* is conidia-specific. Thus, this experiment shows that the product of ApvA can also be produced inside conidia and incorporated into melanin, suggesting that the tissue specific allocation of their products may be due to cell-type specific expression of the genes *atmelA* and *apvA* under regulation of their specific promoters.

### Tissue specific expression of *gfp* occurs when the coding sequence of *gfp* replaces *atmelA* and *apvA*

To test our hypothesis, we generated two mutants (*atmelAp-gfp*, *apvAp-gfp*) in which the coding regions of *apvA* and *atmelA* were replaced by the green fluorescent protein coding sequence (*gfp*), placing *gfp* under control of their specific promoters (Fig. S1Cii†). Both the melanin pathway and aspulvinones biosynthetic pathways are active when *A. terreus* is cultivated on LCMM agar. Under this culture condition, we hypothesized that *atmelAp* would turn on the expression of *gfp* specifically inside the conidia while the *gfp* regulated by *apvAp* would display a green fluorescent signal in the hyphae. As expected, we were able to visualize localization of GFP within the conidia only in the strain carrying *atmelAp-gfp* but not in the parental strain (used as control) or the strain *apvAp-gfp*. (Fig. 5B) In comparison, hyphal localization of GFP fluorescence was observed only in strain *apvAp-gfp*. (Fig. 5B) Thus, the tissue specific accumulation of aspulvinone E (**1**) is probably due to cell-type-specific expression of the two genes *apvA* and *atmelA*.

## Discussion

Our study suggests that metabolite diversity can be expanded by spatial regulation of the same precursor biosynthesized by two distinct genes. Melanin plays an important role in fungal pathogenesis as well as in the protection of the producing organisms from ultraviolet radiation.^11,16,17^ No definite structures for melanins have been elucidated due to their large molecular weight, insolubility in aqueous or organic solvents, and heterogeneity.^11^ The characterized conidial melanin of most *Aspergillus* species belongs to the dihydroxynaphthalene (DHN) melanin class.^11^ Our study reports a unique and unprecedented pathway of conidial pigment biosynthesis in *A. terreus* that originates from the NRPS-like product aspulvinone E (**1**) (Fig. 1). We also identified one putative tyrosinase encoded by the gene *atmelB* that is involved in a tailoring modification of aspulvinone E (**1**) to yield the brown conidial pigment (Fig. 1). We speculate that the function of the tyrosinase, AtmelB, might resemble that of its homolog which participates in dihydroxyphenylalanine (DOPA)-melanin biosynthesis^11^ and catalyzes the *ortho* hydroxylation of the phenol moiety in the aspulvinone E (**1**) core (Fig. 1). It is entirely possible that other genes are involved in the polymerization of the dual hydroxylated aspulvinone E and more efforts will be needed to decipher the complete biosynthetic pathway of the brown conidial melanin in *A. terreus*.

Compounds in the aspulvinone family have been found to have anti-influenza A viral (H1N1) activity and are potent inhibitors of firefly luciferase.^18,19^ The production of aspulvinones, as revealed in a previous study, is a general stress response of *A. terreus*.^20^ The biological functions of aspulvinones, and what role they play in the biology of *A. terreus*, remain elusive. AtmelA shares 73% sequence similarity with ApvA. We noted that there is a 63 bp sequence in *atmelA*, corresponding to the TE domain of AtmelA, that has strong nucleotide identity with a portion of *apvA*, indicating that these two genes may share a common origin (Fig. S5C†). Interestingly, homology analysis of *apvA* and its surrounding genes showed that *apvA* is inserted into a conserved genome region consisting of genes that are essential for fungal survival (Fig. S4†). It is possible that the insertion of *apvA* is due to the duplication and translocation of its ancestral homolog. The production of aspulvinone-type SMs by *A. terreus*, in response to general stress, could be related to the chromosomal location of its producing gene *apvA*. However, more experiments are definitely necessary to elucidate the exact role the aspulvinone-type metabolites play in the growth, survival or metabolic processes of the fungus *A. terreus*.

Previous literature suggested that *p*-hydroxylphenylpyruvate (HPP), which originates from the shikimate pathway, is the biosynthetic precursor of aspulvinones.^21,22^ The shikimate pathway, highly conserved in bacteria, fungi, and plant species, generates carbon skeletons for the aromatic amino acids including tryptophan, tyrosine, and phenylalanine.^23^ HPP is proposed to be an intermediate in the conversion of prephenate to l-tyrosine in the shikimate pathway. Limited information has been reported regarding the tissue localization of HPP in fungi. Our study suggests that the distribution of HPP is not limited to certain types of fungal compartments since both the biosynthesis of brown conidial melanin and hyphae specific aspulvinones requires the presence of HPP. Our data indicate that the tissue-specific expression of *apvA* and *atmelA* are due to the promoters of the two genes. The promoter of *apvA* drives expression specifically in hyphae while the promoter of *atmelA* drives expression in conidia. There are many examples of hyphal *versus* spore specific gene regulation. For example, the expression of the melanin synthesis gene *atmelA* might regulated by spore specific transcriptional regulators as demonstrated in a recent study showing that BrlA, the transcription factor required to initiate conidiophore development in *Aspergillus* spp., is necessary for fumiquinazoline gene expression and product production.^13^ The fact that *atmelAp* is capable of turning on the expression of both *apvA* (SM gene) and the *gfp* (reporter gene), suggests that it might be a useful tool for directing the expression of genes specifically inside of conidia.

Besides spatial regulation of the intermediate, the SM diversity can be further expanded, as shown in our study, through tailoring enzymes that are capable of modifying SMs with different chemical scaffolds. Early literature reported the enzymatic characterization of aspulvinone dimethylallyltransferase in *A. terreus*. This enzyme is capable of catalyzing the mono or dual prenylation of aspulvinone E (**1**).^24^ The substrate promiscuity of some PTs has also been tested *in vitro* by feeding experiments.^25^ Our study reveals the *in vivo* versatility of AbpB, as it accepts substrates with different chemical scaffolds. Interestingly, the three genes are dispersed in the *A. terreus* genome (*abpB* on chromosome II; *apvA* on chromosome III; *btyA* on chromosome IV), representing another deviation from the SM gene cluster paradigm. We have discovered in *A. nidulans* that in some cases, the corresponding PT genes are not located in the same cluster as the core PKS genes.^26,27^ Further comparison of the butyrolactones with aspulvinones shows that the chemical modifications after prenylation, including epoxidation and dehydrogenation, are very similar (Fig. S6†). Thus, these two natural product families may share the same set of tailoring enzymes that specifically modifies the prenyl groups attached by AbpB.

Fungi are capable of producing a large variety of SMs with unique chemical scaffolds. It is usually expected that core synthetic genes, in a given species, are behind the biosynthesis of different core intermediates, as demonstrated in a study showing that all individual NRPKS in *A. nidulans* generate a unique PKS product.^5^ Phylogenetic analysis, using the protein sequences of those NRPS-like homologs obtained from the Broad Institute *Aspergillus* comparative database, revealed several other characterized NRPS-like homologs including TdiA (terrequinone A biosynthesis in *A. nidulans*),^8^ RalA (ralfuranone biosynthesis in *Ralstonia solanacearum*),^28^ MicA (microperfuranone biosynthesis in *A. nidulans*),^29^ AtqA (asterriquinone biosynthesis in *A. terreus*),^7^ BtyA (butyrolactone biosynthesis in *A. terreus*),^7^ and EchA (echosides biosynthesis in *Streptomyces* sp. LZ35)^30^ (Fig. S5†). These NRPS-like enzymes, with A-T-TE domain architecture, fall within Clade I of the phylogenetic tree (Fig. S5†). They are capable of synthesizing natural products with various chemical scaffolds using similar substrates like phenylpyruvic acid. Previous literature suggested that these pyruvic acid substrates could be produced *via* the shikimate pathway.^21,31,32^ The TE domains are proposed to catalyze various condensation, cyclization and releasing reactions to yield different chemical backbones.^8^ In comparison, another characterized NRPS-like protein (encoded by ATEG_03630.1), with A-T-R domain arrangement, belongs to Clade V (Fig. S5†).^33^ In this case, the aryl acid substrate, generated by an adjacent PKS (encoded by ATEG_03629.1), is loaded onto the A domain and is reduced to its aryl-aldehyde precursor by the R domain. Thus, SM diversity is enriched from these disparate starting points. It is also common to identify two highly homologous core synthetic genes, in different species, that produces the same precursor. For example, in the biosynthesis of the meroterpenoids austinol (*A. nidulans*) and terretonin (*A. terreus*), the two PKS genes *ausA* and *trt4* synthesize the same intermediate 3, 5-dimethylorsellinic acid.^34^

## Conclusion

Our work indicates that chemical diversity in SM biosynthesis can be expanded *via* multiple dimensions. Prior to our work, the richness of the fungal SM pool was presumed to be determined by the number of SM gene clusters while the clustered genes are associated with the biosynthesis of a distinct type of SM, with some exceptions.^12,13,35^ In our study, we have shown that *A. terreus* deploys an additional strategy to enrich its natural product pool. Although the same precursor aspulvinone E (**1**) is shared in two pathways (the aspulvinone pathway, a typical SM pathway and the melanin pathway, producing a self-protection pigment that might also be involved in the pathogenicity of this fungus), the tissue specific expression of their biosynthetic genes results in the production of the same compound in different fungal tissues and allows it to be modified into two different products that, we assume, confer selective advantages in the specific tissues in which they are produced. The localization of production is possibly regulated by their specific promoters, but it is entirely possible that a more complex regulatory mechanism underlies this phenomenon. This expands our insight into spatial regulation of SMs in fungi.^36^ More investigation into these promoters might provide a means to the cell-type-directed biosynthesis of SMs or manipulating the location of the expression of some genes responsible for products with interesting biological activities. Finally, our data demonstrate that AbpB prenylates compounds in two pathways revealing that two pathways may share the same tailoring genes. It will be of interest to determine if this characteristic is common to, and specific to, prenyl transferases.

## Materials and methods

### Strains and molecular manipulations

Primers used in this study are listed in Table S1.† The fungal strains used in this study are listed in Table S2.† The scheme for heterologously expressing the NRPS-like genes of *A. terreus* in *A. nidulans* is shown in Fig. S1.† The direct repeat (DR) deletion and *AfpyG* marker recycling experimental design is shown in Fig. S1.† The construction of fusion PCR products, protoplast generation, and transformation were carried out as previously described.^7,9^ The scheme of diagnostic PCR is shown in Fig. S8.†

For real time qRT-PCR, the *A. terreus* wild type strain and the mutant strain (*apvA*Δ, *atmelA*::*apvA*) were cultivated on LCMM agar for mRNA extraction from conidia. The *A. terreus* wild type strain was cultivated in LCMM liquid broth for mRNA extraction from hyphae. Total mRNA was extracted by using the Qiagen RNeasy Plant Mini Kit. The cDNA was made from the equal amount of mRNA. The expression of every gene was analyzed with the ABI 7900HT Fast Real-Time PCR system (see ESI for experimental details†).

### Fermentation and LC-MS analysis

(1) *A. nidulans* strain LO4389 and other HE mutant strains were cultivated at 37 °C in 50 ml LMM liquid medium (6 g l^–1^ NaNO_3_, 0.52 g l^–1^ KCl, 0.52 g l^–1^ MgSO_4_·7H_2_O, 1.52 g l^–1^ KH_2_PO_4_, 20 g l^–1^ lactose supplemented with 1 ml l^–1^ of trace element solution) at 1 × 10^6^ spore per ml per 125 ml flask with shaking at 180 rpm. The nutrients uridine, uracil, pyridoxine and riboflavin were supplemented if necessary. As previously reported,^9^ 44 μl (10 mM) cyclopentanone was added into the medium after 18 h of incubation. The incubator temperature was then changed to 30 °C and the culture medium was collected 72 h after cyclopentanone induction. The medium was filtrated and extracted twice with ethyl acetate (EtOAc, 50 ml). Preparation of the HPLC-MS samples and the condition for MS analysis were as previously described.^7^

(2) For the prenyltransferase screening experiment, *A. terreus* NIH 2624 and the mutant strains were point inoculated at 30 °C on LCMM agar plates (6 g l^–1^ NaNO_3_, 0.52 g l^–1^ KCl, 0.52 g l^–1^ MgSO_4_·7H_2_O, 1.52 g l^–1^ KH_2_PO_4_, 10 g l^–1^ d-glucose, 20 g l^–1^ lactose, 15 g l^–1^ agar supplemented with 1 ml l^–1^ of trace element solution) per plate (*D* = 10 cm). After 5 days, agar was chopped into small pieces and extracted with 50 ml MeOH followed by 60 ml 1 : 1 CH_2_Cl_2_/MeOH. The extract was evaporated *in vacuo* to yield a water residue, which was suspended in 25 ml H_2_O and partitioned with 25 ml EtOAc, twice. Preparation of the HPLC-MS samples and the condition for MS analysis were the same as previously described.^7^

(3) For the extraction of different fungal tissues for aspulvinone production, the procedure was similar to a previously reported procedure.^12,13^ Plates containing 20 ml LCMM agar (1.5% agar) were overlaid with another 10 ml of LCMM agar (0.75% agar). Plates were point inoculated with wild type or the DR mutant strains and grown at 30 °C for 4 days. Conidia were harvested by adding 7 ml salt solution (8.5 g l^–1^ NaCl) followed by gently scraping with a sterile spreader. The conidial solution was inspected under the microscope to be largely free of hyphae and conidiophores. The conidial fraction was sonicated for 1 h and extracted twice with an equal volume of EtOAc. The top layer was then washed with 10 ml sterile water twice and removed with a sterile spatula. The bottom agar layer was then chopped into small pieces and extracted as previously described. Preparation of the HPLC-MS samples from different tissues and the conditions for MS analysis were as previously described.^7^

### Isolation of secondary metabolites

For scale up, *A. nidulans* HE strains were cultivated at 37 °C in 1 liter LMM liquid medium (∼100 ml per flask) at 1 × 10^6^ spores per ml with shaking at 180 rpm. The nutrients uridine, uracil, pyridoxine and riboflavin were supplemented if necessary. To induce expression, 88 μl cyclopentanone was added into the medium after 18 h of incubation. The incubator temperature was then changed to 30 °C and the culture medium was collected 72 h after cyclopentanone induction. The medium was filtrated and extracted three times with equal volume of EtOAc. The combined EtOAc layers were evaporated to a crude extract. Further purification of fractions with targeted compounds was carried out by gradient HPLC on a C18 reverse phase column [Phenomenex Luna 5 μm C18, 250 × 10 mm] with a flow rate of 5.0 ml min^–1^ and measured by a UV detector at 254 nm.

### Fluorescence microscopy

The wild type and two mutant strains (*atmelAp-gfp*, *apvAp-gfp*) were cultivated on the two-layer agar plates as mentioned. Plates were point inoculated with wild type or the mutant strains and grown at 30 °C for 5 days. The conidia were collected as previously described and the conidial solution was diluted 10 times with sterile water. A 10 μl portion of the diluted solution was placed on a pre-cleaned microscope slide and covered with a coverslip. For imaging of the hyphal GFP fluorescence signal, the top layer was removed as previously described. A small portion of hyphae containing agar (<10 μl) was placed on a pre-cleaned microscope slide and covered with a coverslip. All the samples were examined under an optical microscope to confirm that the conidia and hyphae could be clearly visualized. Images were taken with a Zeiss LSM 510 Meta NLO (Thornwood, NY) confocal imaging system equipped with Argon, red HeNe, and green HeNe lasers and a Coherent Chameleon Ti–Sapphire laser mounted on a vibration-free table.

## References

[cit1] Khaldi N., Seifuddin F. T., Turner G., Haft D., Nierman W. C., Wolfe K. H., Fedorova N. D. (2010). Fungal Genet. Biol..

[cit2] Keller N. P., Turner G., Bennett J. W. (2005). Nat. Rev. Microbiol..

[cit3] Fischbach M. A., Walsh C. T. (2006). Chem. Rev..

[cit4] Brown D. W., Yu J. H., Kelkar H. S., Fernandes M., Nesbitt T. C., Keller N. P., Adams T. H., Leonard T. J. (1996). Proc. Natl. Acad. Sci. U. S. A..

[cit5] Ahuja M., Chiang Y.-M., Chang S.-L., Praseuth M. B., Entwistle R., Sanchez J. F., Lo H.-C., Yeh H.-H., Oakley B. R., Wang C. C. C. (2012). J. Am. Chem. Soc..

[cit6] von Döhren H. (2009). Fungal Genet. Biol..

[cit7] Guo C. J., Knox B. P., Sanchez J. F., Chiang Y. M., Bruno K. S., Wang C. C. (2013). Org. Lett..

[cit8] Balibar C. J., Howard-Jones A. R., Walsh C. T. (2007). Nat. Chem. Biol..

[cit9] Chiang Y. M., Oakley C. E., Ahuja M., Entwistle R., Schultz A., Chang S. L., Sung C. T., Wang C. C., Oakley B. R. (2013). J. Am. Chem. Soc..

[cit10] Nielsen J. B., Nielsen M. L., Mortensen U. H. (2008). Fungal Genet. Biol..

[cit11] Langfelder K., Streibel M., Jahn B., Haase G., Brakhage A. A. (2003). Fungal Genet. Biol..

[cit12] Berthier E., Lim F. Y., Deng Q., Guo C. J., Kontoyiannis D. P., Wang C. C., Rindy J., Beebe D. J., Huttenlocher A., Keller N. P. (2013). PLoS Pathog..

[cit13] Lim F. Y., Ames B., Walsh C. T., Keller N. P. (2014). Cell. Microbiol..

[cit14] May G. S. (1989). J. Cell Biol..

[cit15] Oakley B. R. (2004). Fungal Genet. Biol..

[cit16] Eisenman H. C., Casadevall A. (2012). Appl. Microbiol. Biotechnol..

[cit17] Gao Q., Garcia-Pichel F. (2011). Nat. Rev. Microbiol..

[cit18] Gao H., Guo W., Wang Q., Zhang L., Zhu M., Zhu T., Gu Q., Wang W., Li D. (2013). Bioorg. Med. Chem. Lett..

[cit19] Cruz P. G., Auld D. S., Schultz P. J., Lovell S., Battaile K. P., MacArthur R., Shen M., Tamayo-Castillo G., Inglese J., Sherman D. H. (2011). Chem. Biol..

[cit20] Hanlon A. and O'Connor S., 2006, http://hdl.handle.net/1721.1/37692.

[cit21] Nitta K., Fujita N., Yoshimura T., Arai K., Yamamoto Y. (1983). Chem. Pharm. Bull..

[cit22] Dewick P. M. (1984). Nat. Prod. Rep..

[cit23] Tohge T., Watanabe M., Hoefgen R., Fernie A. R. (2013). Front. Plant Sci..

[cit24] Takahashi I., Ojima N., Ogura K., Seto S. (1978). Biochemistry.

[cit25] Yu X., Xie X., Li S. M. (2011). Appl. Microbiol. Biotechnol..

[cit26] Sanchez J. F., Entwistle R., Hung J.-H., Yaegashi J., Jain S., Chiang Y.-M., Wang C. C. C., Oakley B. R. (2011). J. Am. Chem. Soc..

[cit27] Lo H.-C., Entwistle R., Guo C.-J., Ahuja M., Szewczyk E., Hung J.-H., Chiang Y.-M., Oakley B. R., Wang C. C. C. (2012). J. Am. Chem. Soc..

[cit28] Wackler B., Schneider P., Jacobs J. M., Pauly J., Allen C., Nett M., Hoffmeister D. (2011). Chem. Biol..

[cit29] Yeh H.-H., Chiang Y.-M., Entwistle R., Ahuja M., Lee K.-H., Bruno K., Wu T.-K., Oakley B., Wang C. C. (2012). Appl. Microbiol. Biotechnol..

[cit30] Zhu J., Chen W., Li Y.-Y., Deng J.-J., Zhu D.-Y., Duan J., Liu Y., Shi G.-Y., Xie C., Wang H.-X., Shen Y.-M. (2014). Gene.

[cit31] Knaggs A. R. (2003). Nat. Prod. Rep..

[cit32] Arai K., Yamamoto Y. (1990). Chem. Pharm. Bull..

[cit33] Wang M., Beissner M., Zhao H. (2014). Chem. Biol..

[cit34] Guo C.-J., Knox B. P., Chiang Y.-M., Lo H.-C., Sanchez J. F., Lee K.-H., Oakley B. R., Bruno K. S., Wang C. C. C. (2012). Org. Lett..

[cit35] Wiemann P., Guo C. J., Palmer J. M., Sekonyela R., Wang C. C., Keller N. P. (2013). Proc. Natl. Acad. Sci. U. S. A..

[cit36] Lim F. Y., Keller N. P. (2014). Nat. Prod. Rep..

