# Supplementary material for: Spatial regulation of a common precursor from two distinct genes generates metabolite diversity
Source: Chem Sci. 2015 Jul 13;6(10):5913–21. doi: 10.1039/c5sc01058f (PMC5523082; doi:10.1039/c5sc01058f)
Supplement: Supplementary file 1 [file SC-006-C5SC01058F-s001.pdf]

Electronic Supplementary Material (ESI) for Chemical Science.
This journal is © The Royal Society of Chemistry 2015

Supporting Information

Spatial Regulation of a Common Precursor from Two Distinct Genes Generates Metabolite Diversity

Chun-Jun Guo, Wei-Wen Sun, Kenneth S Bruno, Berl R. Oakley, Nancy P. Keller, and Clay C. C. Wang

Correspondence should be addressed to C.C.C.W. (clayw@usc.edu).

Table of Contents

Supplemental Methods S2

Spectral Data of compounds S2

Table S1. Primers used in this study S3

Table S2. *A. terreus* strains used in this study S6

Table S3. NMR data for compound **1** S7

Table S4. NMR data for compound **5** S8

Figure S1. The schematic design of molecular genetic experiments performed in this study: (A) heterologous expression of *A. terreus* NRPS-like genes in *A. nidulans*; (B) direct repeat experiments; (C) gene replacement and *gfp* replacement experiments. S9

Figure S2. Relative quantification analysis of gene expression levels in the *A.terreus* wild type-hyphae, wild type-conidia, and CW6058.1 (*apvA*Δ, *atmelA*::*apvA*) -conidia. S11

Figure S3. HPLC profiles of extracts of wild type and the *apvA*Δ, *atmelA::apvA* strain as detected by UV at 370nm. S12

Figure S4. The gene *apvA* (red arrow) is inserted in a highly conserved region among *Aspergillus* species that contains genes putatively encoding life-essential proteins. S12

Figure S5. Homology analysis of the NRPS-like homologs in *Aspergillus* species. S13

Figure S6. Proposed biosynthetic pathway for butyrolactones and aspulvinones. S15

Figure S7. UV-Vis and ESIMS spectra of compounds **1**, **5**. S16

Figure S8. Diagnostic PCR strategies. S17

Figure S9-S10. NMR spectra of compounds **1** and **5**. S19-S20

Supplemental references S21

Supplemental methods

Isolation of secondary metabolites

The gradient system was MeCN (solvent B) and 5% MeCN/H_2O (solvent A) both containing 0.05% TFA. Compound **1** was identified in both the secondary metabolites profiles of the *alcA_apvA* and *alcA_atmelA* mutant strains. The gradient condition for semi-preparative HPLC analysis of the crude of the *alcA_atmelA* strain was 0-2 min 100%-70% A, 2-5 min 70% A, 15-17 min 70%-0% A, 17-19 min 0%-100% A, 19-21 min 100% A. Compounds **1** (76.17 mg/L of medium) was eluted at 13.1 min.

Real-Time qRT-PCR analysis of the expression of genes *atmelA, apvA, abpB,* and *btyA*.

The *A. terreus* wild type and the mutant strain CW6058.1 (*apvA*Δ, *atmelAp-apvA*) were cultivated on LCMM agar at 30 ℃ for 72 hours for extracting mRNA from spores. The *A. terreus* wild type was cultured in LCMM broth (1M spores/ml of medium) at 37 ℃, 180rpm, for 60 hours for hyphal mRNA extraction. The β-tubulin gene *atbenA* (ATEG_00287.1) was used as a control and quantification standard. Total mRNA was extracted by using the Qiagen RNeasy Plant Mini Kit. The total mRNA was digested by Recombinant DNase I (ambion by life technologies) to remove DNA contamination. The cDNA library was made from the same amount of mRNA by using TaqMan reverse transcription reagents (T04141) and the oligo DT primer. The expression of every gene was analyzed with the ABI 7900HT Fast Real-Time PCR system by following the KAPA SYBR FAST qPCR kit (KK4601) protocol. The experiments were performed in triplicate and the results are shown in Figure S2.

Spectral data of Compounds

NMR spectra were collected on a Varian Mercury Plus 400 spectrometer. The spectral data of compounds **2**, **3** and **4** have been reported before.

Aspulvinone E (**1**).Yellowish amorphous solid; For UV-Vis and ESIMS spectra, see Figure S6; For NMR spectra, see Table S3. The NMR data were in good agreement with the published data. **Butyrolactone II** (**5**). Colorless amorphous solid; For UV-Vis and ESIMS spectra, see Figure S6; For NMR spectra, see Table S4.

Table S1. Primers used in this study

primer	Sequence (5′→3′)
Primers used in the heterologous expression experiments	
ATEG2004.1HEF	CCA ATC CTA TCA CCT CGC CTC AAA ATG ACT TTG AAC AAC CTA CA
ATEG2004.1HER	CGA AGA GGG TGA AGA GCA TTG CGC TTG ACT TTC AAT AGA CG
ATEG3563.1HEF	CCA ATC CTA TCA CCT CGC CTC AAA ATG CAA CCA AGC CTT ATT CC
ATEG3563.1HER	CGA AGA GGG TGA AGA GCA TTG TTC CTC GAG AGT TTG AGA A
Primer used in the prenyltransferase deletion experiments	
ATEG_00702.1F1	GTT ATG TTG GCC TCG AGA TG
ATEG_00702.1F2	GGC CAT TTT GTA ATG CTG TC
ATEG_00702.1R3	CGA AGA GGG TGA AGA GCA TTG AAG GTC TCA TCG GAG AGG AT
ATEG_00702.1F4	CAT CAG TGC CTC CTC TCA GAC AGC ATA ATG ACC ATC CGC TTG
ATEG_00702.1R5	ATG AAG GTC GCT CGT GTT AC
ATEG_00702.1R6	TTC TTC CAT TCC TCA CCA TC
ATEG_00821.1F1	GTA AAG GCC AAT GAA GAT GG
ATEG_00821.1F2	TAG TCC GAA TCC TCC CAT AG
ATEG_00821.1R3	CGA AGA GGG TGA AGA GCA TTG GAG GAC AAA TAG CCA GAT CG
ATEG_00821.1F4	CAT CAG TGC CTC CTC TCA GAC AGT TTA CCG GGT ATT CCA TCT G
ATEG_00821.1R5	ATC TGT TGA AGC GGC ATA GT
ATEG_00821.1R6	AAA CGC CAG TAC GAA TCT GT
ATEG_01730.1F1	ATT CTG CAT TTG GTC CTA CG
ATEG_01730.1F2	TCT CCA AGT AAG GAG CCA GA
ATEG_01730.1R3	CGA AGA GGG TGA AGA GCA TTG GGA AGA AAC GAT TCT GAT GC
ATEG_01730.1F4	CAT CAG TGC CTC CTC TCA GAC AGA GTG CTC CTT CAT CAC GTC T
ATEG_01730.1R5	GGA CAT CGA TTG TCT CAA CC
ATEG_01730.1R6	CTT TGT GTA CCA AGG CCA AG
ATEG_02823.1F1	GGG TTG GCA TCA AAC TCA
ATEG_02823.1F2	GGG ATG TCA TTC CAC AGT TC
ATEG_02823.1R3	CGA AGA GGG TGA AGA GCA TTG CGT ATG ACC TGG AGG TGA AG
ATEG_02823.1F4	CAT CAG TGC CTC CTC TCA GAC AGA GAG ACC CCC ATT TCA ATT C
ATEG_02823.1R5	GTC ATT GAT CCG TGC AAA G
ATEG_02823.1R6	TGA ATC GTT GCA GTA GTT CG
ATEG_03092.1F1	AGA AGT TGC CAT CGA AGT TG
ATEG_03092.1F2	GGG TTT TTG TAC TTG GTG CT
ATEG_03092.1R3	CGA AGA GGG TGA AGA GCA TTG GGT GGT AGT CGG TGA TAA GC
ATEG_03092.1F4	CAT CAG TGC CTC CTC TCA GAC AGA TCA GGT TCT GCA GTT ACG G
ATEG_03092.1R5	ATT CGG CCG TGT TCT CAT AC
ATEG_03092.1R6	TCC AAC TCC TAC CTT CAT CG
ATEG_04218.1F1	GCC CTA CTC TGA TCC TGA CA
ATEG_04218.1F2	CAT GGC CAA AGA CAA AAG AC
ATEG_04218.1R3	CGA AGA GGG TGA AGA GCA TTG TAT GCT TGA TGG CAG GAT G
ATEG_04218.1F4	CAT CAG TGC CTC CTC TCA GAC AGG AGC AGT AGG TTT GCA GGA C
ATEG_04218.1R5	GTC GGG TTC TGA GGG TTA CT
ATEG_04218.1R6	ATG ATG ATT CCG TGC TGA C
ATEG_04999.1F1	TCA GTG TGG ATG CAG GAT AG
ATEG_04999.1F2	GGT TGC TTC CAT TAT GTC GT
ATEG_04999.1R3	CGA AGA GGG TGA AGA GCA TTG GAG TCG ATG GGA TGT CAA GT
ATEG_04999.1F4	CAT CAG TGC CTC CTC TCA GAC AGT GAC TCT TGT ACT GGG TTT CC
ATEG_04999.1R5	CAC ATC TCC AAC AAC CAT CA

ATEG_04999.1R6	ATC TCG CTC ACA TCT CCA AC
ATEG_06111.1F1	GCT TCC ATG TCG AAC TGT G
ATEG_06111.1F2	CGA GTA CAT CTG TTG GTA GGC
ATEG_06111.1R3	CGA AGA GGG TGA AGA GCA TTG GAG GAG GTA CTG CTG GAA AA
ATEG_06111.1F4	CAT CAG TGC CTC CTC TCA GAC AGG TGT TAT ACT GGA GCC ACT GC
ATEG_06111.1R5	CAG GGC TAA TGC GTT ATT GT
ATEG_06111.1R6	GAC AGA CTC GAT GGA TGG TT
ATEG_06825.1F1	ATT CAG CCT CTC ATT GAA GC
ATEG_06825.1F2	GTA TCA CGA GAC CCA AAA CC
ATEG_06825.1R3	CGA AGA GGG TGA AGA GCA TTG TAG AAT GCA TGT TCG TCG AG
ATEG_06825.1F4	CAT CAG TGC CTC CTC TCA GAC AGC ATA GAG CGC TGC AAA TGT A
ATEG_06825.1R5	TGC TAC TGA CGA AAG TGG TC
ATEG_06825.1R6	ATC CGC GAC TAT GCT ACT GA
ATEG_08428.1F1	AAT TCA CCG AGA CAA CAT CC
ATEG_08428.1F2	GTT GGG TGT ATC AGG GAA GA
ATEG_08428.1R3	CGA AGA GGG TGA AGA GCA TTG ATG CTG TGT AAC ACG GAT TG
ATEG_08428.1F4	CAT CAG TGC CTC CTC TCA GAC AGC CAA GAG CTC AGT CGT TCA
ATEG_08428.1R5	ATC GCA GAG CTT CAG TCA TT
ATEG_08428.1R6	GTA TCC AAT CGC AGA GCT TC
ATEG_09980.1F1	CTG AAA AAT GAG CGG AGA AG
ATEG_09980.1F2	GGC AAA TCT GCC TGT TAG AC
ATEG_09980.1R3	CGA AGA GGG TGA AGA GCA TTG TGG TCG AAT ATG GGA CTA GC
ATEG_09980.1F4	CAT CAG TGC CTC CTC TCA GAC AGG GTA TGG GTT GCC AGA TAG A
ATEG_09980.1R5	GGC GAG CTG TAC TTC ATC A
ATEG_09980.1R6	AGA GTC GTC GCT GTA GGT GT
ATEG_10306.1F1	CTC GTG CAG GTT TAA CGA AC
ATEG_10306.1F2	CGT TAA TGT TCC TTG GGT GA
ATEG_10306.1R3	CGA AGA GGG TGA AGA GCA TTG GTG GAA GGG GAA ATG GTT AT
ATEG_10306.1F4	CAT CAG TGC CTC CTC TCA GAC AGA AGA TGA ATC GTG GCA GTG T
ATEG_10306.1R5	AGG GCT TAC AAT GGA TGC TA
ATEG_10306.1R6	TGG CCA ATG TAG GTA GAA GC
Primer used in the direct repeat (DR) disruption experiments	
ATEG2004.1DR_F1	ATT ATG TAG CAG CAC GCA AG
ATEG2004.1DR_F2	GGT ATG GAT CGT TTC GTG TT
ATEG2004.1DR_R3	GAC AAA TTC CCG AGA AAC AGG CTG ATC ATG AAG ATG CTT G
ATEG2004.1DR_F4	CTG TTT CTC GGG AAT TTG TC
ATEG2004.1DR_R5	CGA AGA GGG TGA AGA GCA TTG TTA CTG CTG TCG ACT TCG TG
ATEG2004.1DR_R6	GTG ATT GTC GGC CAG AAT AG
ATEG3564.1DR_F1	CCA TGG AGA AGA AGA CCA AG
ATEG3564.1DR_F2	GTT AAC AAG CAC CAT TCT ACC C
ATEG3564.1DR_R3	TAC TCT TTG TGG TTT ACC GGT CAC GCA GTG AAG TCA TCA T
ATEG3564.1DR_F4	CCG GTA AAC CAC AAA GAG TA
ATEG3564.1DR_R5	CGA AGA GGG TGA AGA GCA TTG TGA GAC TGA AGA CGC TGA AG
ATEG3564.1DR_F6	CAT CAG TGC CTC CTC TCA GAC AGA GTT CCC GGT AAA CCA CAA
ATEG3564.1DR_R7	GAT AGT GAA CAC AGC GAG GA
ATEG3564.1DR_R8	GGC TGA AGA GGA TAG TGA ACA
ATEG3563.1DR_F1	CGT CGC TCA AAT GAC TTA GA
ATEG3563.1DR_F2	AGA GTC TTC TCC GTG GTC TG
ATEG3563.1DR_R3	ATT ACC ACC CGT AGA GTC GAA CCC TGT ACA TCC TGG AAA A
ATEG3563.1DR_F4	TCG ACT CTA CGG GTG GTA AT

ATEG3563.1DR_R5	CGA AGA GGG TGA AGA GCA TTG ATC TGT GCT GTG CCA TGA TA
ATEG3563.1DR_R6	GAG ACT CGT CTC TCG AGC TT
*AfpyrG*_DR_F1	CGG CGG CTT CTA TTT TAG AA
*AfpyrG*_DR_R2	GGA AGA GAG GTT CAC ACC (M2 primer(1))
AfpyrG_DR_R3	CAG TGC CTC CTC TCA GAC AG
*AfpyrG*_DR_F4	TGA TAC AGG TCT CGG TCC (M3 primer(1))
Primers used in the gene swap experiments	
ATEG3563.1_SWA_F1	ATEG3563.1DR_F1
ATEG3563.1_SWA_F2	ATEG3563.1DR_F2
ATEG3563.1_SWA_R3	TGT AGG TTG TTC AAA GTC ATG GTG TGA TGA AGA AAT CCC C
ATEG3563.1_SWA_F4	CAT CAG TGC CTC CTC TCA GAC AGT CGA CTC TAC GGG TGG TAA T
ATEG3563.1_SWA_R5	CTC GAG CTT ATC TTC CCT GT
ATEG3563.1_SWA_R6	GAG ACT CGT CTC TCG AGC TT
Primers used in the green fluorescent (GFP) experiments	
ATEG3563_GFP_F1	GGG GAT TTC TTC ATC ACA CCA TGA GTA AAG GAG AAG AAC T
ATEG2004_GFP_F1	CCC TTA TTG CAA CTC GGA CCA AAA ATG AGT AAA GGA GAA G
GFP_R2	CGA AGA GGG TGA AGA GCA TTG TTT GAG GCG ACC GGT TTA TTT G
Primers used in the real-time qRT-PCR	
ATEG0287_RT_F	CTT CTC CGT CGT TCC CTC TC
ATEG0287_RT_R	GAG GGG TTG GAG AGC TTG AG
ATEG3563_RT_F	CAT CTG GGT TTT GCG GAT GC
ATEG3563_RT_R	TGC GGC TGT TTG GAT TTG AC
ATEG2004_RT_F	GAT CAT GCT GAT GAC GCA CA
ATEG2004_RT_R	GGT CAA CGA TAT ACT GGG CGA
ATEG1730_RT_F	ACC GAA TCC TCA CGC ATC AA
ATEG1730_RT_R	ACA ATA TGG GCT GGA CAC GG
ATEG2815_RT_F	CAG CAC GGT AAG GAC GAA GT
ATEG2815_RT_R	TTC TGG ATT GCT CTG GGC TG
Primer used in the diagnostic PCR	
*AfpyrG*_R	CGG GAG CAG CGT AGA TGC C

Table S2. Fungal strains used in this study

Fungal strain or transformants	Gene mutation(s)	Genotype
Aspergillus terreus NIH2624	-	wild-type
LO4389 (*A. nidulans*)	None	*pyrG89*; *pyroA4*; *nkuA*::*argB*; *riboB2*; *stcA-W*
CW6050.1, CW6050.2, CW6050.3	*stcJ*Δ, *alcA*(p)-*apvA*	*pyrG89*; *pyroA4*; *nkuA*::*argB*; *riboB2*; *stcA-W* *wA*::*alcA*(p)-*apvA-AfpyrG*
CW6052.1, CW6052.2, CW6052.3	*stcJ*Δ, *alcA*(p)-*atmelA*	*pyrG89*; *pyroA4*; *nkuA*::*argB*; *riboB2*; *stcA-W* *wA*::*alcA*(p)-*atmelA-AfpyrG*
CW6054.1, CW6054.2, CW6054.3	*abpB*Δ	*kusA*:: *hph*; *pyrG-*, *abpB*Δ
CW6055.1, CW6055.2, CW6055.3	*apvA*Δ	*kusA*:: *hph*; *pyrG-*, *apvA*Δ
CW6056.1, CW6056.2, CW6056.3	*apvA*Δ, *atmelB*Δ	*kusA*:: *hph*; *pyrG-*, *apvA*Δ, *atmelB*Δ
CW6057.1, CW6057.2, CW6057.3	*apvA*Δ, *atmelB*Δ, *atmelA*Δ	*kusA*:: *hph*; *pyrG-*, *apvA*Δ, *atmelB*Δ, *atmelA*Δ
CW6058.1, CW6058.2, CW6058.3	*apvA*Δ, *atmelAp-apvA*	*kusA*:: *hph*; *pyrG-*, *apvA*Δ, *atmelAp-apvA-AfpyrG*
CW6059.1, CW6059.2, CW6059.3	*atmelAp-gfp*	*kusA*:: *hph*; *pyrG-*, *atmelAp-gfp-AfpyrG*
CW6060.1, CW6060.2, CW6060.3	*apvAp-gfp*	*kusA*:: *hph*; *pyrG-*, *apvAp-gfp-AfpyrG*

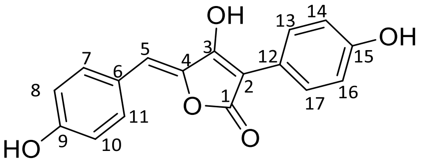

Aspulvinone E

Table S3. ^{1}H and ^{13}C NMR data for compound **1** (400 MHz and 100 MHz in DMSO-d_6) (2)

Position	δ H (*J* in Hz)	δ C
1		168.5, C
2		1001., C
3		162.1, C
4		140.4, C
5	6.64, s	107.8, CH
6		124.2, C
7	7.61, d (7.6)	132.2, CH
8	6.88, d (8.0)	116.2, CH
9		158.5, C
10	6.88, d (8.0)	116.2, CH
11	7.61, d (7.6)	132.2, CH
12		121.0, C
13	7.81, d (8.0)	128.8, CH
14	6.86, d (8.8)	115.4, CH
15		156.7, C
16	6.86, d (8.8)	115.4, CH
17	7.81, d (8.0)	128.8, CH

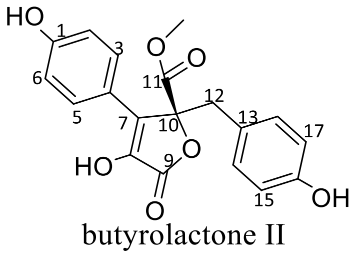

butyrolactone II

Table S4. NMR data for compound **5** (400 and 100 MHz in DMSO-d_6)

Position	δH (J in Hz)	δC
1		158.0, C
2	6.88, d (6.8)	115.9, CH
3	7.52, d (7.2)	128.8, CH
4		121.0, C
5	7.52, d (7.2)	128.8, CH
6	6.88, d (6.8)	115.9, CH
7		127.5, C
8		138.1, C
9		168.0, C
10		84.7, C
11		169.8, C
12	3.40, d (3.2)	38.0, CH_2
13		123.2 C
14	6.58, d (6.8)	131.2, CH
15	6.51, d (6.8)	114.7, CH
16	—	156.3, C
17	6.51, d (6.8)	114.7, CH
18	6.58, d (6.8)	131.2, CH
11-OCH_3	3.74, s	53.6, CH_3

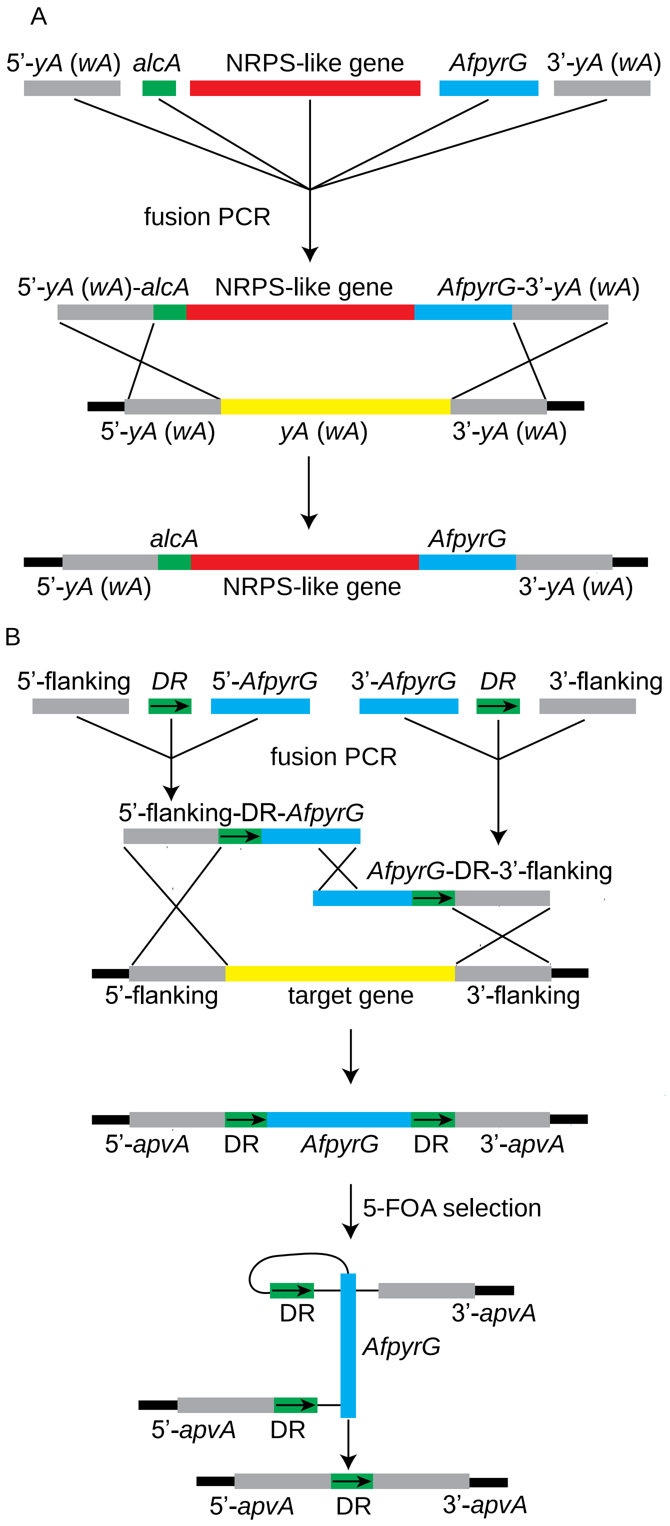
A
5'-yA (wA)
alcA
NRPS-like gene
AfpyrG
3'-yA (wA)
fusion PCR
5'-yA (wA)-alcA
NRPS-like gene
AfpyrG-3'-yA (wA)
5'-yA (wA)
yA (wA)
3'-yA (wA)
alcA
AfpyrG
5'-yA (wA)
NRPS-like gene
3'-yA (wA)
B
5'-flanking
DR
5'-AfpyrG
3'-AfpyrG
DR
3'-flanking
fusion PCR
5'-flanking-DR-AfpyrG
AfpyrG-DR-3'-flanking
5'-flanking
target gene
3'-flanking
5'-apvA
DR
AfpyrG
DR
3'-apvA
5-FOA selection
DR
3'-apvA
AfpyrG
5'-apvA
DR
5'-apvA
DR
3'-apvA

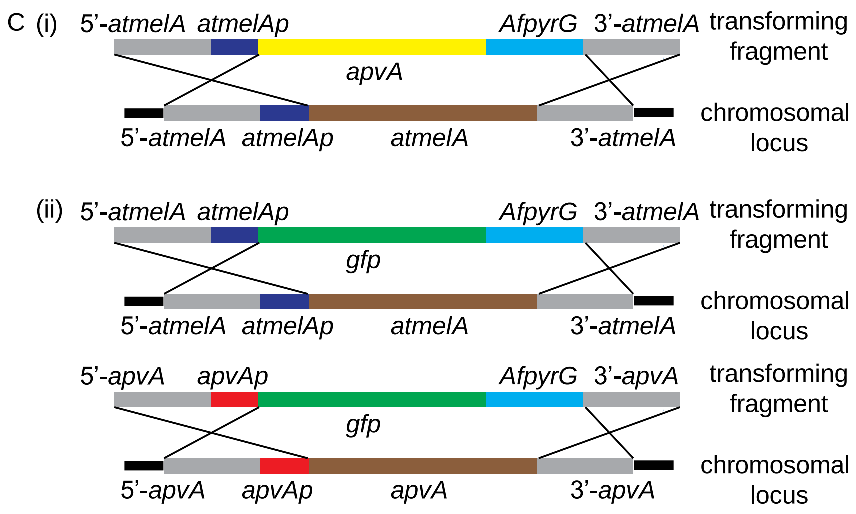

Figure S1. The schematic design of molecular genetic experiments in this study.
(A) Heterologous expression of *A. terreus* NRPS-like genes in *A. nidulans*. The target strain is transformed with a fragment containing the NRPS-like gene and the *A. fumigatus pyrG* gene (*AfpyrG*) flanked by two 1 kb sequences.
(B) Direct repeat (DR) deletion experiments. The target strain is transformed with two fragments: one contains the 5'-flanking sequence, the DR strand, and a partial sequence of *AfpyrG*; the other fragment contains a partial sequence of *AfpyrG*, the DR strand, and the 3'-flanking sequence. In the next round of selection, homologous recombination of the DR strand generates an auxotrophic mutant that can be used in the next transformation.
(C) i. the gene *atmelA* is replaced by *apvA* under the control of the *atmelA* promoter; ii. the coding regions of both *atmelA* and *apvA* are replaced by *gfp* under the control of their own promoters.

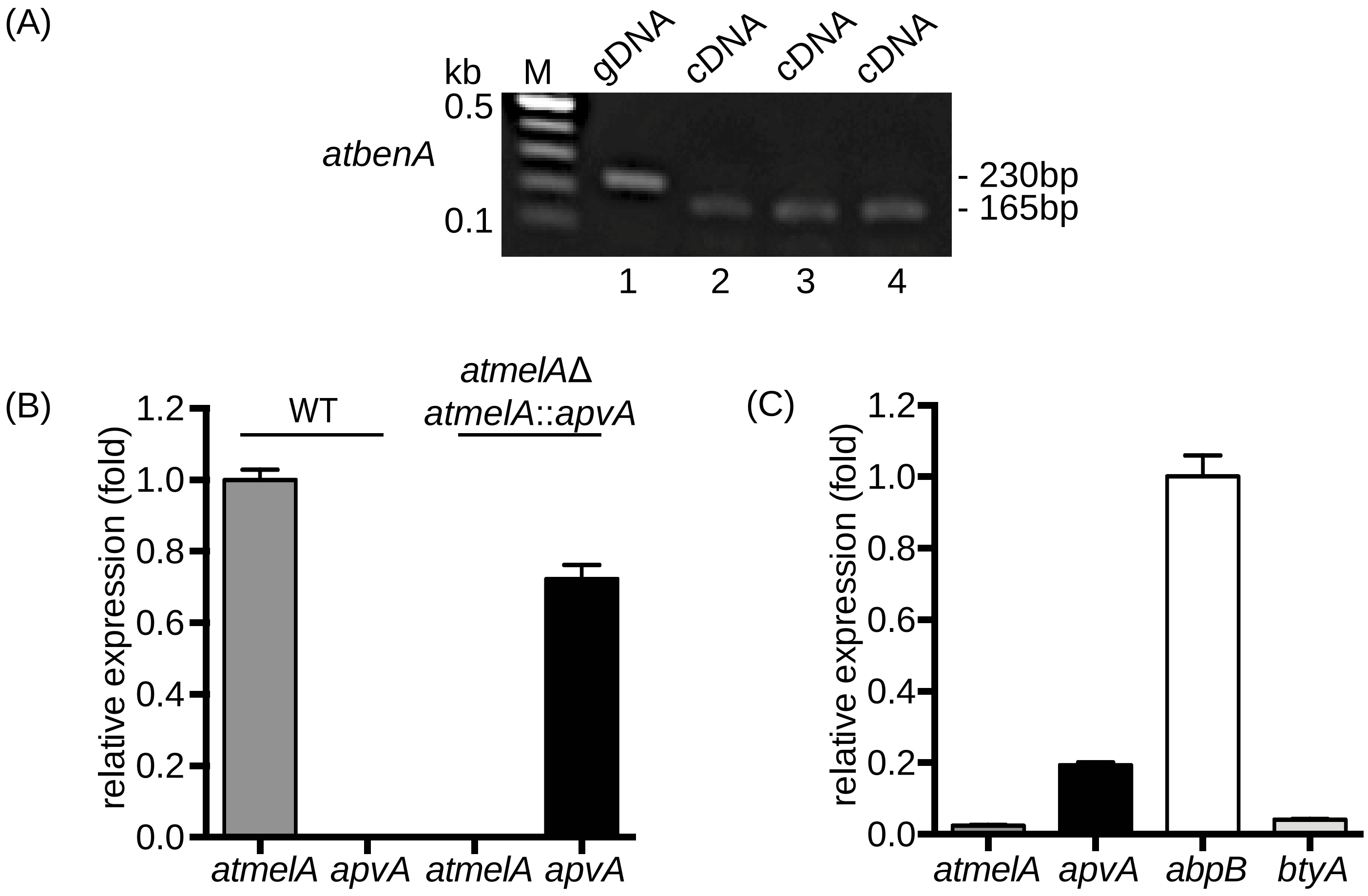

Figure S2. Relative quantification analysis of gene expression levels in the *A.terreus* wild type-hyphae, wild type-conidia, and CW6058.1 (*apvA*Δ, *atmelA*::*apvA*) -conidia. (A) The size of Real-Time PCR product of the β-tubulin gene *atbenA* from cDNA was analyzed using the genomic DNA as control. The mRNAs were extracted from wild type-hyphae (A, lane 2), wild type-conidia (A, lane 3), and CW6058.1-conidia (A, lane 4). (B) The relative expression level of *atmelA* and *apvA* in wild type-conidia and CW6058.1-conidia. (C) The relative expression level of *atmelA*, *apvA*, *abpB,* and *btyA* in wild type-hyphae. Minor expression of *atmelA* can still be identified due to the conidiation (although suppressed in liquid culture) and melanin production of the wild type strain in LCMM liquid broth. The gene expression levels are normalized to *atbenA* in the corresponding cDNA sample. Relative expression levels were calculated using the $2^{-\Delta\Delta Ct}$ method.

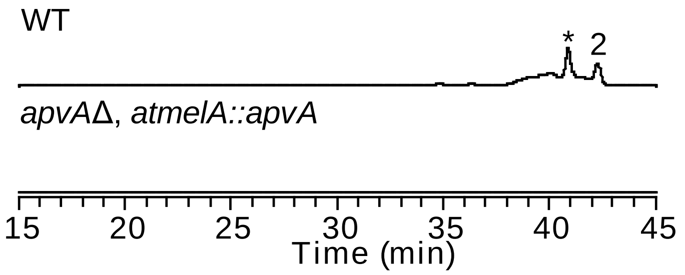

Figure S3. HPLC profiles of extracts of wild type and the *apvA*Δ, *atmelA::apvA* strain as detected by UV at 370nm. The "*" compound is same as shown in Figure 4.

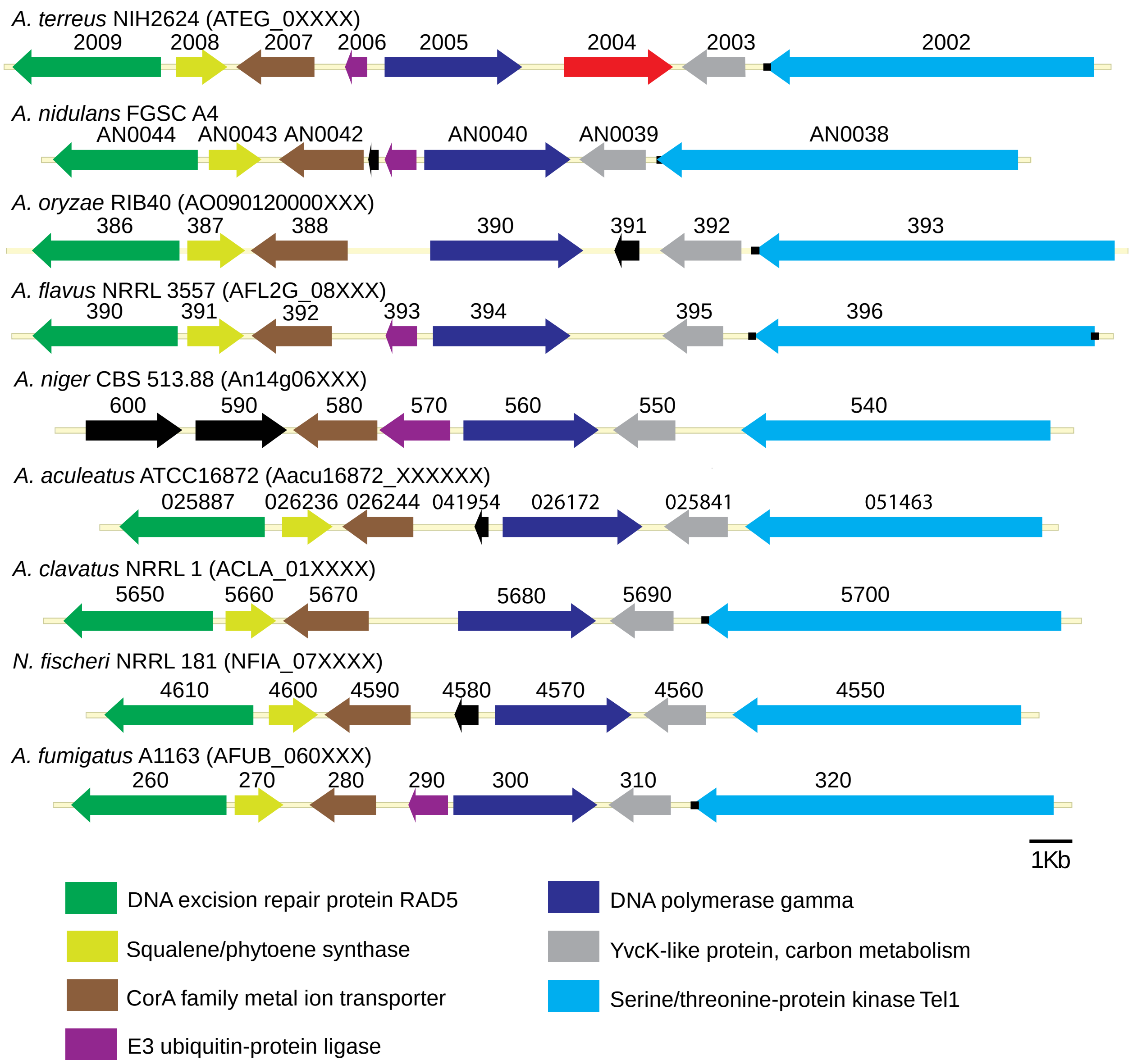

Figure S4. The gene *apvA* (red arrow) is inserted in a highly conserved region among *Aspergillus* species that contains genes putatively encoding life-essential proteins.

(A)

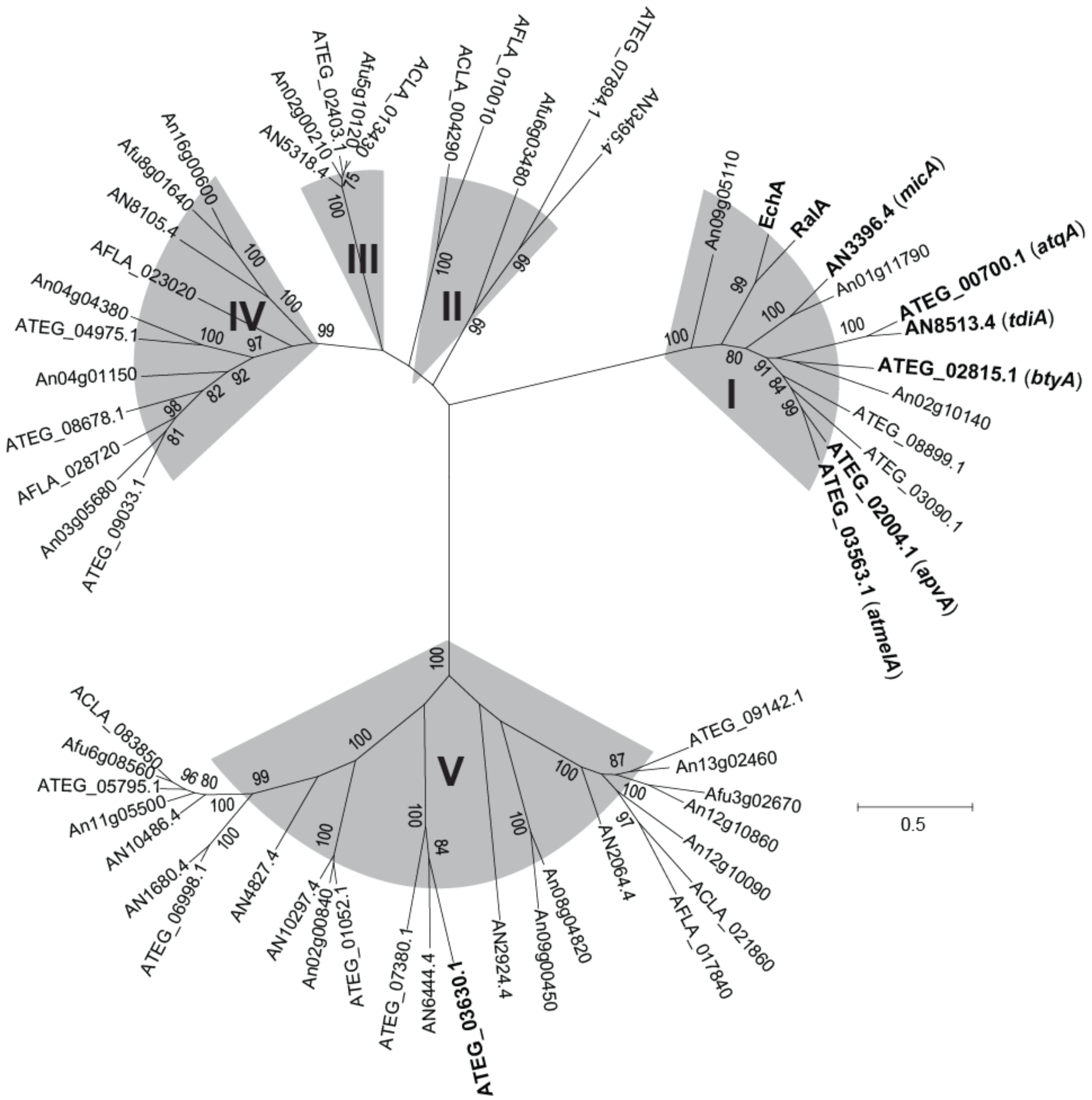

(B)

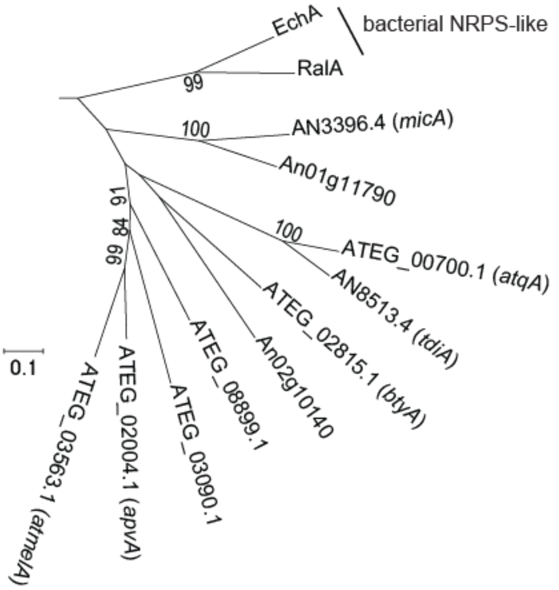

(C)

atmelA 5'-ATGCGCATGCGCGAGCTGGATTGGAAAGAAT

apvA 5'-ATGCGCATGCGCGAGCTGGACTGGAAGGAAT

GCCTGCTCCATCTAGCGTACTTCCTAGATCTG-3'

GCCTGCTCCACCTGGCGTACTTCCTGGATCTG-3'

Figure S5. (A) Homology analysis of 59 NRPS-like homologs obtained from the Broad Institute *Aspergillus* Comparative Database. Phylogenetic analyses of the protein sequences of all the NRPS-like genes identified in *Aspergillus* species. A Maximum Likelihood phylogenetic tree is drawn to scale, with branch lengths in the same units as those of the evolutionary distances used to infer the tree. The numbers on the branches means the percentage of times this topology was reached in a bootstrap test of 1000 replicates. The characterized genes are shown in bold. Genes start with "ACLA" are from the genome sequence of *A.clavatus* ("AFLA", *A. flavus*; "Afu", *A. fumigatus*; "AN", *A. nidulans*. "An", *A. niger*; "ATEG", *A. terreus*;) The percentage lower than 75% is removed. (B). The enlarged part of Clade I of the phylogenetic tree including all the characterzied NRPS-like genes. These genes encode proteins with A-T-TE domain architecture and usually the aryl acids are the subsrate of their A domains. (C) The conserved nucleotide region identified within the two genes *atmelA* and *apvA*.

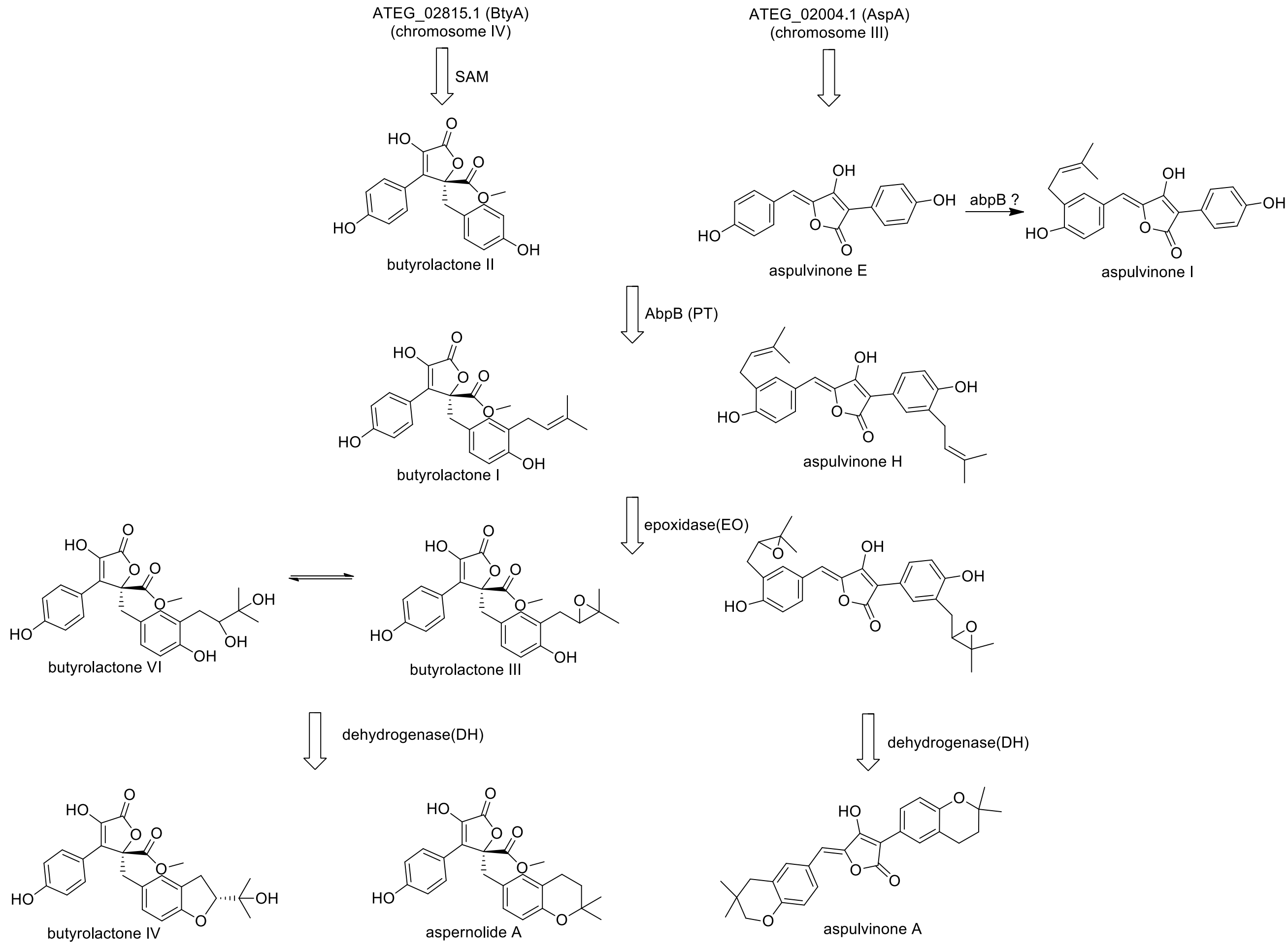

Figure S6. Proposed biosynthetic pathway for butyrolactones and aspulvinones.

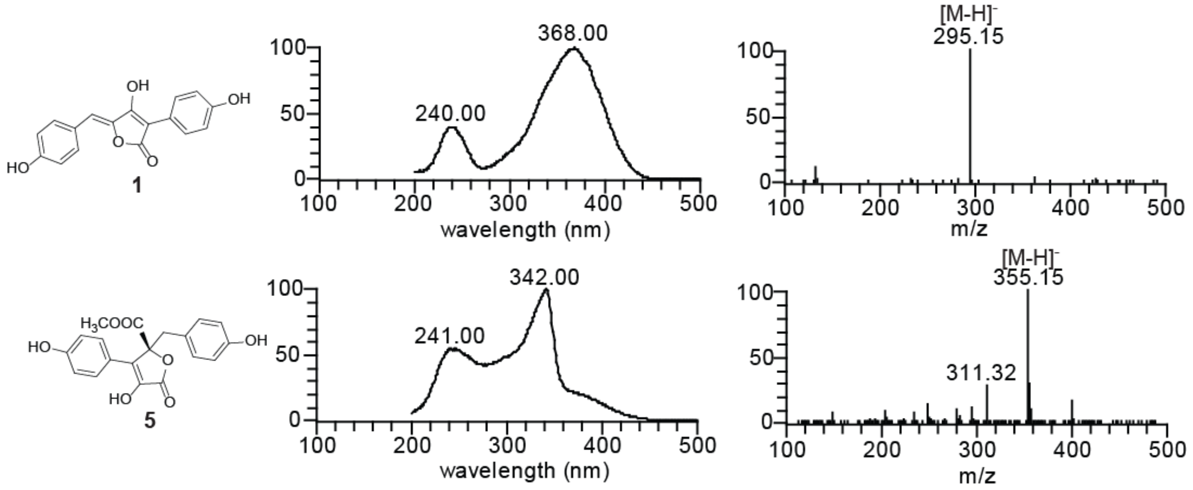

Figure S7. UV-Vis and ESIMS spectra of compounds **1**, **5**.

The UV-Vis and ESIMS spectra of compounds **2**, **3**, and **4** have been shown in the previous paper.(3)

Figure S8. Diagnostic PCR strategies.

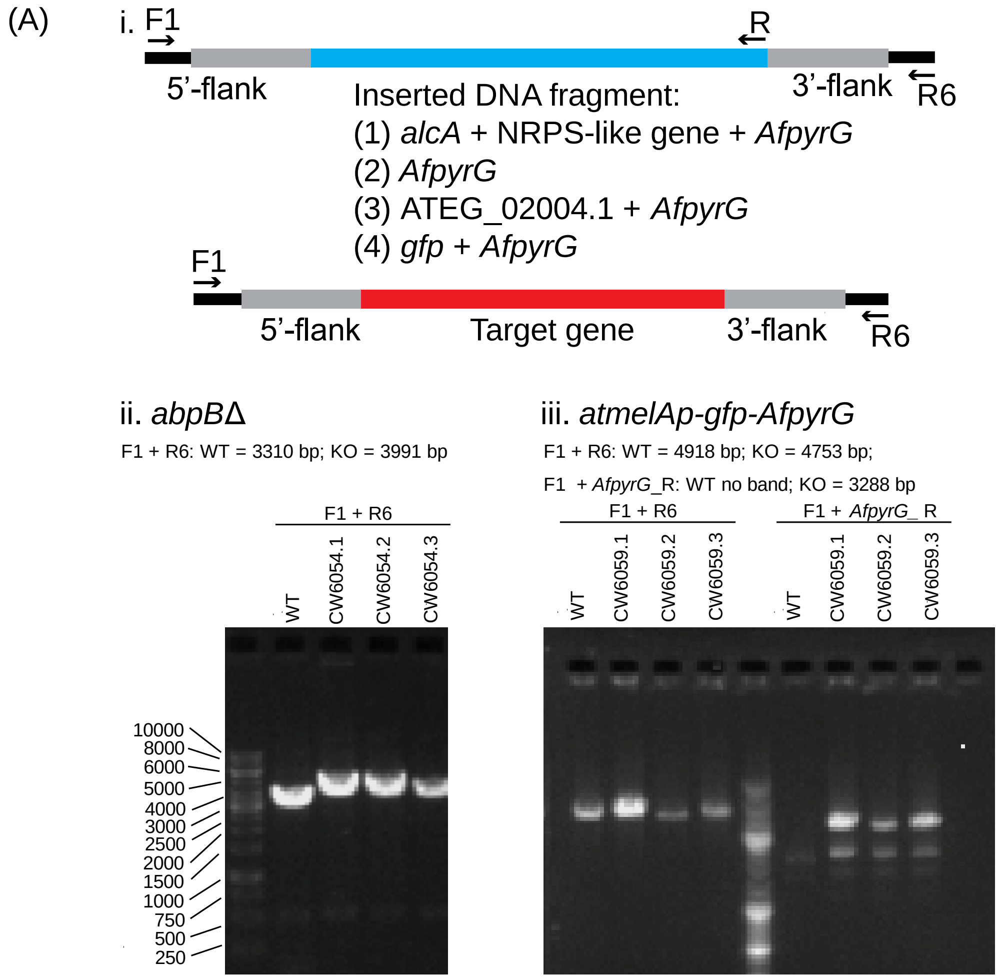

(A) Diagnostic PCR for NRPS-like genes heterologous expression (1), PT gene deletions (2), gene swap experiment (3), and *gfp* replacement experiment (4) (number corresponds to the fragment that is inserted in each experiment). In one strategy, DNA from transformants is amplified with two primers, F1 from the chromosomal region just outside of the 5' flank of the transforming DNA fragment and R6 from just outside of the 3' flank. If the target gene is different in size from the inserted fragment, the PCR fragment amplified from a correct transformant will be different in size from the fragment amplified if the target gene is intact, as shown in the case of *abpB*Δ (A ii). In some instances the target gene and the *AfpyrG* cassette will be of comparable size and a second strategy is applied. In the second strategy, F1 or R6 are used with internal primers specific to the *AfpyrG* cassette. For example, if the target gene has been replaced by the *AfpyrG* gene, F1 and *AfpyrG*R will amplify a fragment of a predictable size. If the target gene has not been replaced, the *AfpyrG*R primer will not anneal and there will be no specific amplification, as shown in the case of mutant strain *atmelAp-gfp-AfpyrG* (A iii).

(B) i.

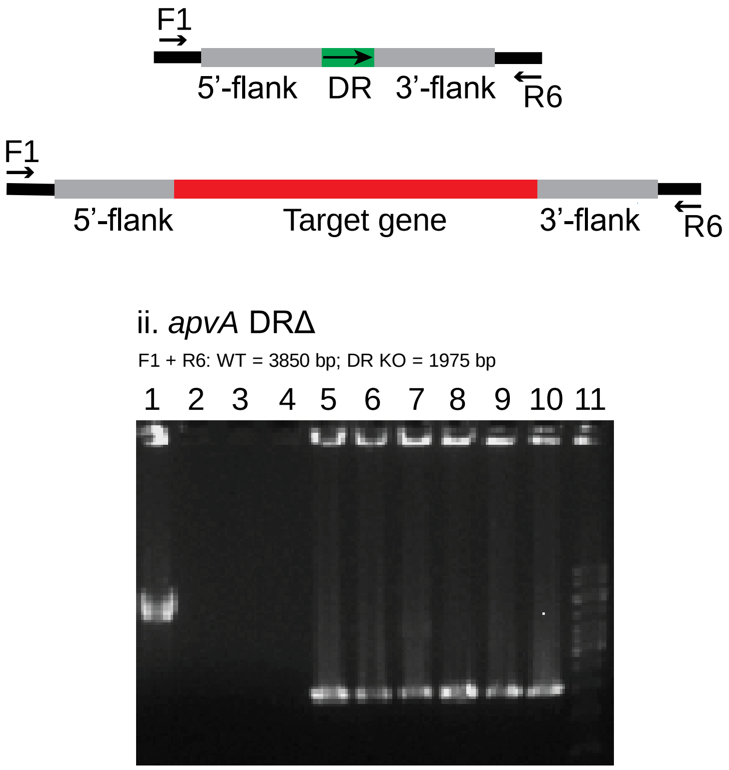

(B) Diagnostic PCR for direct repeat experiment

For direct repeat (DR) experiment, the diagnostic PCR experiments were performed after the *AfpyrG* marker has been cut off via homologous recombination of the DR sequences since the PCR experiments using F1 and R6 cannot be performed when there are two copies of DR sequence integrated in the genome (C). An example of diagnostic PCR for DR mutant strains is shown in B ii. Lane 1 shows PCR amplification from the wild type strain. Lanes 5-10 show PCR amplification from the *apvA* DR deletion mutants when the *AfpryG* marker has been excised.

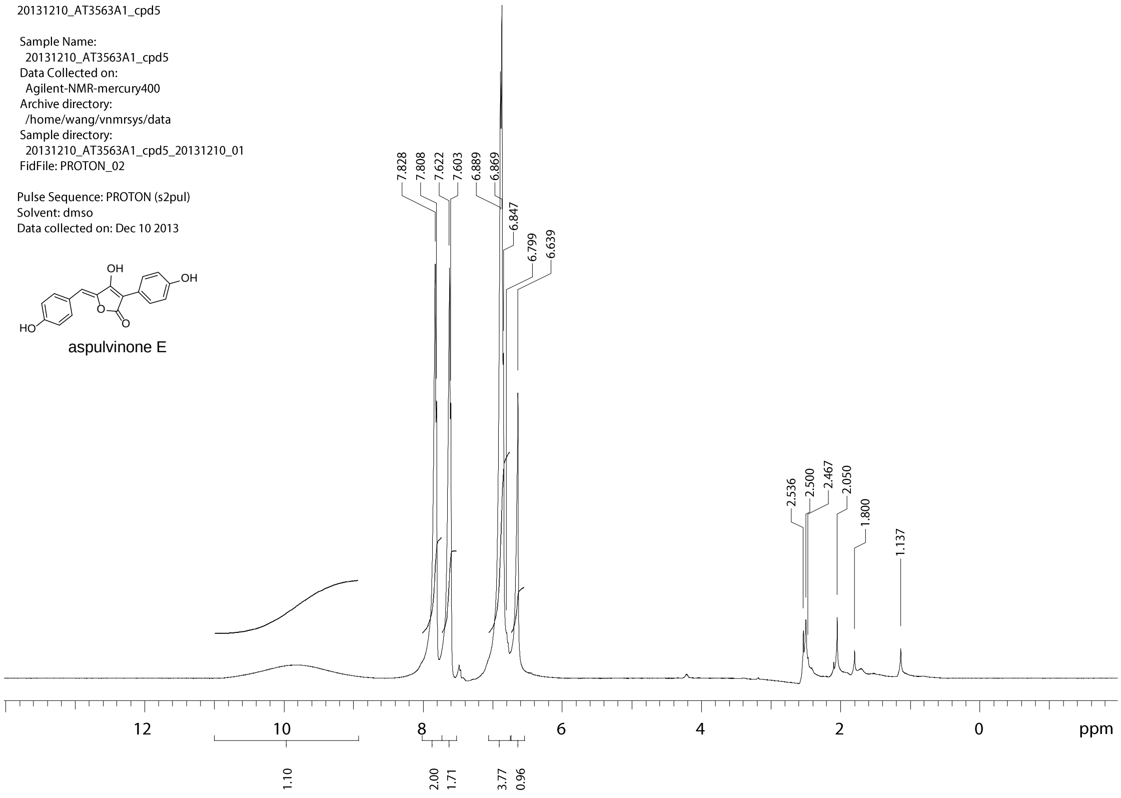

a. ^{1}H NMR spectrum of compound **1**

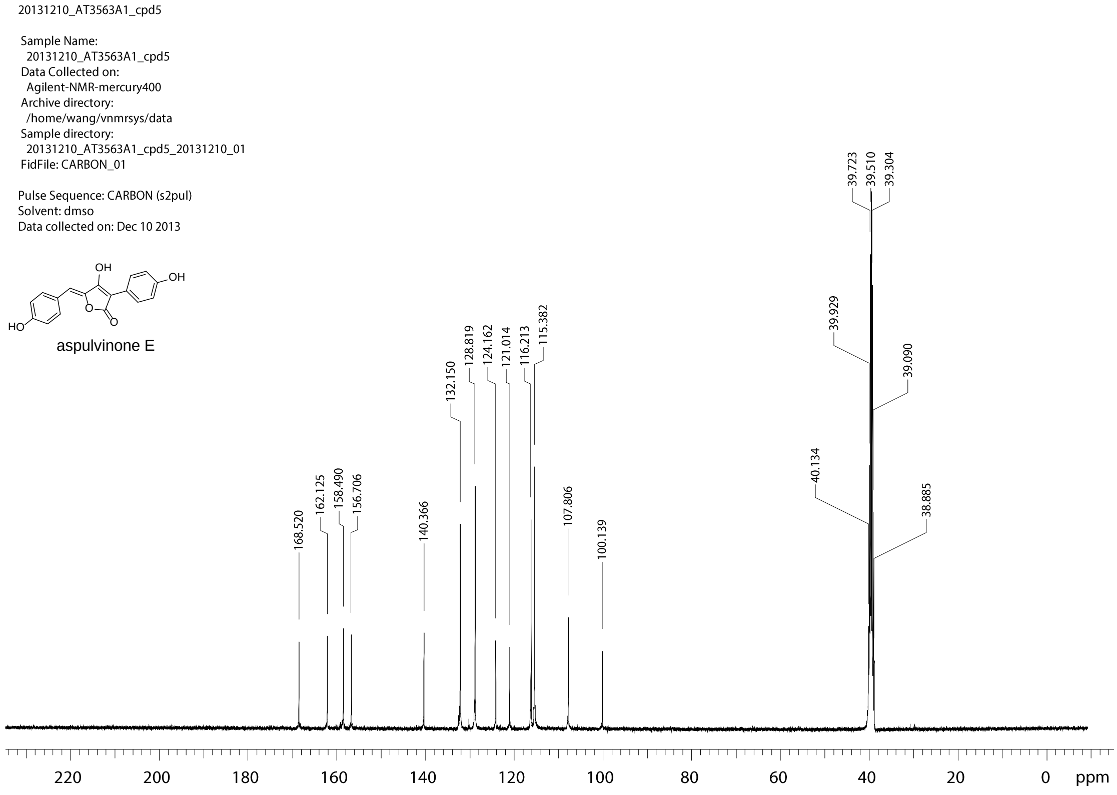

b.^{13}C NMR spectrum of compound **1**

Figure S9. ^{1}H NMR and ^{13}C spectra of compound **1**.

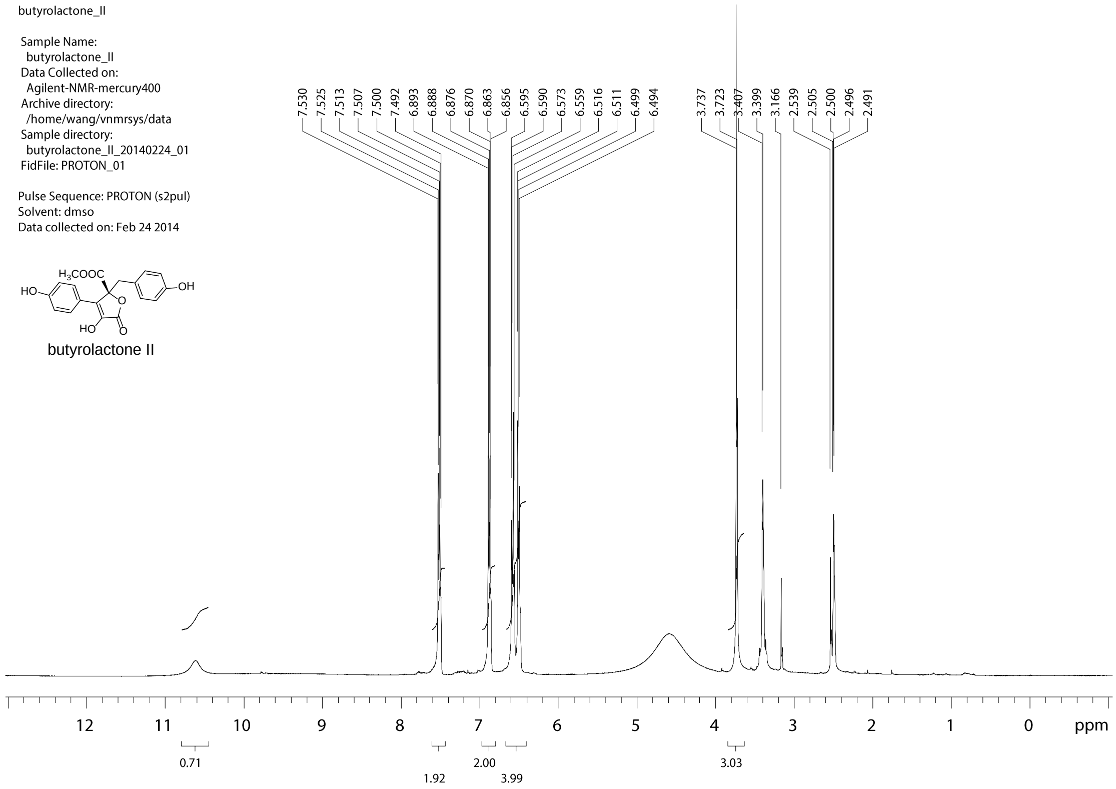

a.^{1}H NMR spectrum of compound **5**

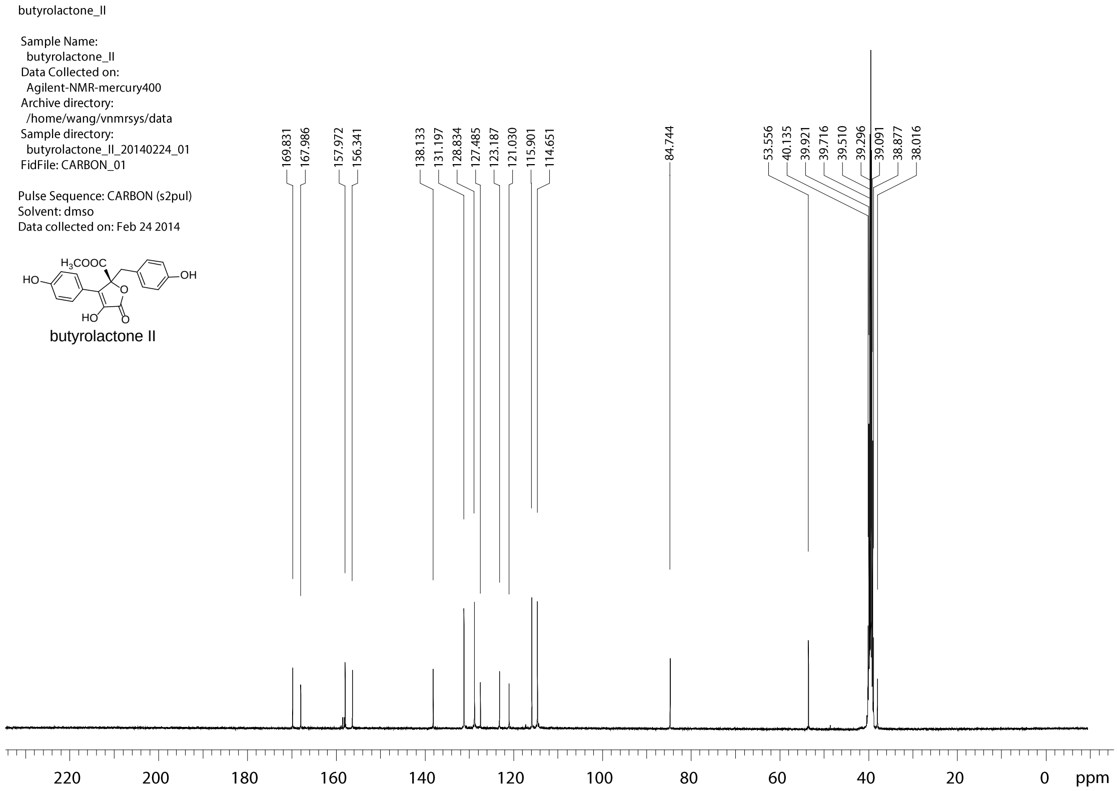

b.^{13}C NMR spectrum of compound **5**

Figure S10. ^{1}H NMR and ^{13}C spectra of compound **5**.

Supplemental References

1.Nielsen JB, Nielsen ML, & Mortensen UH (2008) Transient disruption of non-homologous end-joining facilitates targeted genome manipulations in the filamentous fungus *Aspergillus nidulans*. *Fungal Genet Biol* 45(3):165-170.

2.Gao H, *et al.* (2013) Aspulvinones from a mangrove rhizosphere soil-derived fungus *Aspergillus terreus* Gwq-48 with anti-influenza A viral (H1N1) activity*Bioorg Med Chem Lett* 23(6):1776-1778.

3.Guo CJ, *et al.* (2013) Application of an efficient gene targeting system linking secondary metabolites to their biosynthetic genes in *Aspergillus terreus*. *Org Lett* 15(14):3562-3565.
